# 14-3-3ζ promotes hepatocellular carcinoma venous metastasis by modulating hypoxia-inducible factor-1α

**DOI:** 10.18632/oncotarget.7493

**Published:** 2016-02-19

**Authors:** Yufu Tang, Shupeng Liu, Nan Li, Weixing Guo, Jie Shi, Hongming Yu, Long Zhang, Kang Wang, Shangrong Liu, Shuqun Cheng

**Affiliations:** ^1^ Eastern Hepatobiliary Surgery Hospital, Second Military Medical University, Shanghai 200438, China; ^2^ Changhai Hospital, Second Military Medical University, Shanghai 200433, China; ^3^ Department of Hepatobiliary Surgery, General Hospital of Shenyang Military Area Command, Liaoning 110016, China

**Keywords:** hypoxia, portal vein tumor thrombus, hepatocellular carcinoma, metastasis

## Abstract

Portal vein tumor thrombus (PVTT) is a type of intrahepatic metastasis arising from hepatocellular carcinoma (HCC) and is highly correlated with a poor prognosis. Hypoxia is common in solider tumors, including HCC, where it alters the behavior of HCC cells. We asked whether and how hypoxia contributes to PVTT formation. We demonstrated that increased intratumoral hypoxia is strongly associated with PVTT formation in HCC. We also showed that 14-3-3ζ is induced by hypoxia in HCC cells and correlates with PVTT formation in clinical HCC samples. In addition, 14-3-3ζ up-regulates HIF-1α expression by recruiting HDAC4, which prevents HIF-1α acetylation, thereby stabilizing the protein. Under hypoxic conditions *in vitro*, 14-3-3ζ knockdown inhibits hypoxia-induced HCC invasion by the HIF-1α/EMT pathway. Blockade of 14-3-3ζ in HCC cells reduces PVTT formation and distant lung metastasis *in vivo*. Moreover, a combination of 14-3-3ζ and HIF-1α expression is more prognostic for HCC patients than either protein alone. These results suggest that the hypoxia/14-3-3ζ/HIF-1α pathway plays an important role in PVTT formation and HCC metastasis.

## INTRODUCTION

Hepatocellular carcinoma (HCC) is the fifth most common cancer worldwide and the third leading cause of death from cancer worldwide [1, 2]. Both intra- and extrahepatic metastases result in a poor prognosis for HCC patients [3, 4]. Portal vein tumor thrombus (PVTT), arising from the invasion of HCC cells into the portal vein (trunk or branch), is a special type of intrahepatic metastasis of HCC [5]. Approximately 20%–70% of HCC is accompanied by PVTT as determined by autopsy and/or radiographic examination, while up to 90% of HCC patients have PVTT determined by microscopic examination [6]. HCC patients with PVTT exhibited a poorer clinical outcome [5, 7]. Clarification of the mechanisms underlying the formation and development of PVTT is crucial for developing novel therapeutic strategies for HCC patients.

Hypoxia is common in tumors including HCC. Hypoxia alters HCC cell proliferation, apoptosis, metastasis, chemoresistance, and radioresistance [8]. In HCC, hypoxia results from a shortage of blood circulation caused by liver cirrhosis and the rapid growth of tumor cells. Interestingly, liver cirrhosis and tumor size (> 8 cm) are both independent predictors of PVTT in HCC [9]. In addition, expression of protein disulfide-isomerase A6 (PDIA6), apolipoprotein A-I (APO A-I) and CXC chemokine receptor 4 (CXCR4), correlate with PVTT, and can also be induced by hypoxia [10, 11]. Thus, PVTT formation may be associated with the hypoxic microenvironment of HCC. However, there is no direct evidence showing that hypoxia contributes to the formation of PVTT.

In the present study, we uncovered a causative link between intratumoral hypoxia and PVTT formation. Hypoxia increased the expression of 14-3-3ζ, which induced hypoxia-induced factor-1α (HIF-1α) expression by stabilizing HIF-1α protein. This resulted in an enhanced EMT response of HCC cells, promoting the formation of PVTT and HCC metastasis. These results reveal that the hypoxia/14-3-3ζ/HIF-1α pathway underlies PVTT formation and may contribute to the development of new therapeutics for HCC patients.

## RESULTS

### Increased intratumoral hypoxia is associated with PVTT formation in HCC patients

**Figure 1:**
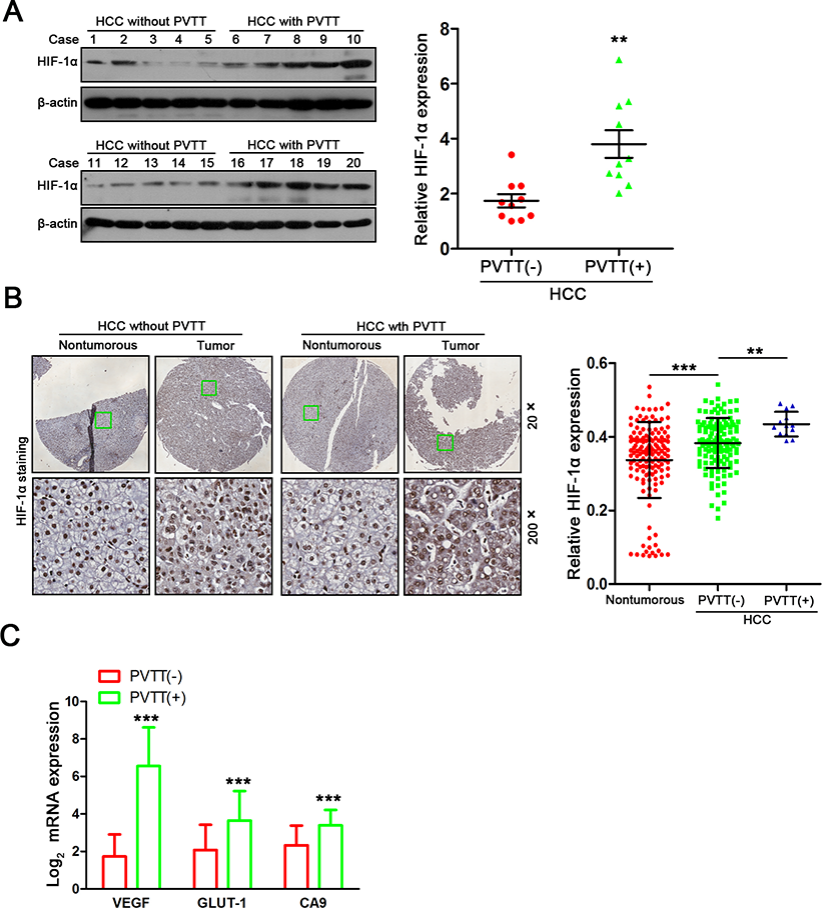
Increased intratumoral hypoxia is associated with PVTT formation in HCC patients (**A**) HIF-1α expression in 10 PVTT(−) HCC tumor samples and 10 PVTT(+) HCC tumor samples were evaluated by western blot. **P < 0.01. (**B**) Relative HIF-1α expression in normal liver tissues, PVTT(−) HCC tissues and PVTT(+) HCC tissues among 143 HCC samples. Representative IHC images are shown in (left panel). **P < 0.01. (**C**) The mRNA expression levels of target genes (VEGF, GLUT-1, and CA9) were detected using quantitative real time PCR in 20 PVTT(−) HCC tumor samples and 20 PVTT(+)HCC tumor samples. **P < 0.01.

To investigate whether hypoxia is associated with the formation of PVTT, HIF-1α, a key factor regulates cellular responses to hypoxia [12], was first examined in primary HCC samples from patients with ([PVTT(+)], *n* = 10) or without ([PVTT(−)], *n* = 10) PVTT by western blot. As shown in Figure 1A, higher expression of HIF-1α was observed in PVTT(+) primary HCC samples compared with PVTT(−) primary HCC samples. We next performed immunohistochemical (IHC) analysis on HCC tissue microarrays (TMAs) containing 143 paired normal/HCC samples. PVTT(+) primary HCC samples had a higher density of HIF-1α protein staining than PVTT(−) or normal liver samples (Figure 1B). To assess HIF-1α transcriptional activity, we measured the expression of HIF-1α-dependent genes: vascular endothelial growth factor A (*VEGF-A*), glucose transporter 1 (*GLUT-1*), and carbonic anhydrase 9 (*CA9*) in 20 PVTT(−) and 20 PVTT(+) primary HCC samples. All three of the target genes were more highly expressed in PVTT(+) HCC samples (Figure 1C). These results suggest that intratumoral hypoxia/HIF-1α is positively correlated with the formation of PVTT.

### Both HIF-1α levels and PVTT formation in HCC are strongly correlated with 14-3-3ζ expression

**Figure 2:**
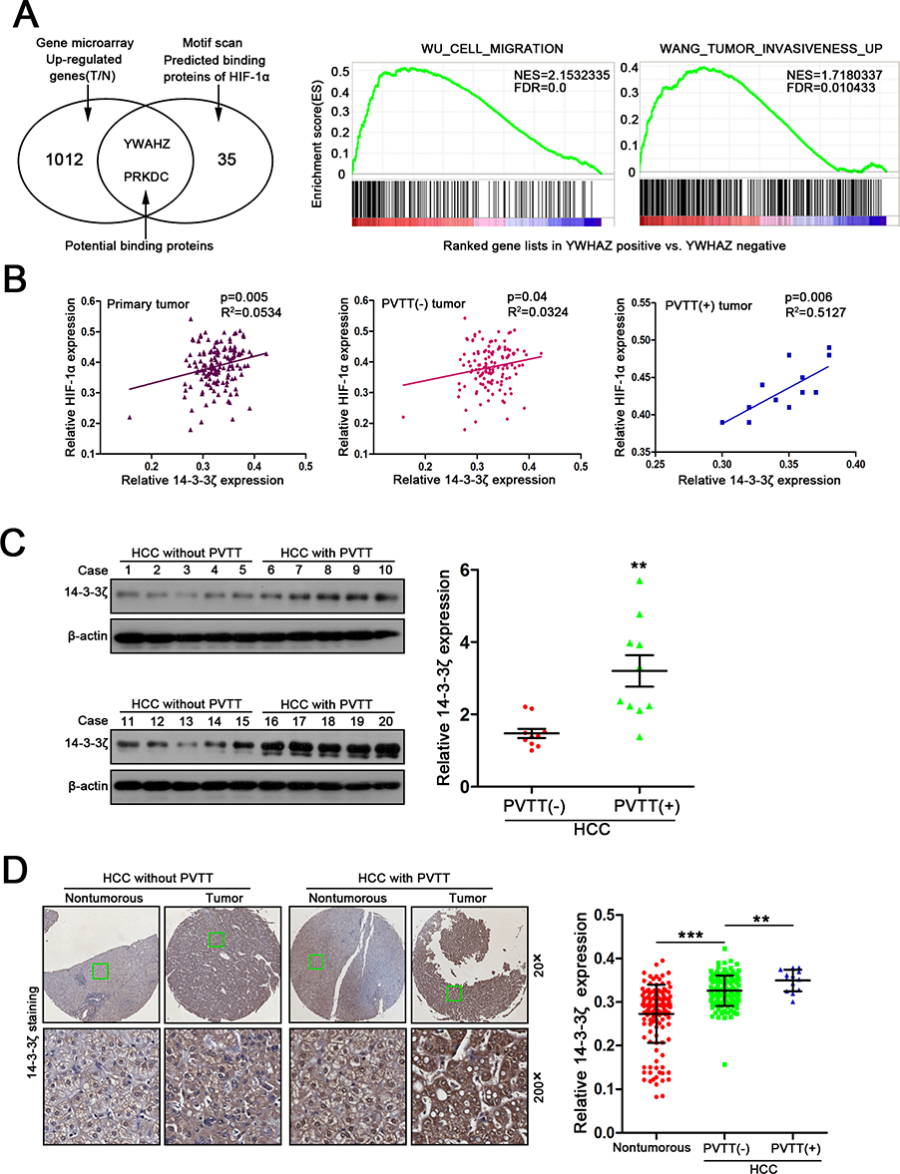
14-3-3ζ expression is strongly correlated with HIF-1α expression and the formation of PVTT in HCC (**A**) Bioinformatic analysis of HIF-1α related genes (left panel, venn diagram) and 14-3-3ζ related pathways involved in invasion and metastasis. (**B**) Correlation between HIF-1α expression and 14-3-3ζ expression was examined in total 144 tumor tissues (*R*^2^ = 0.0534, *P* = 0.005), PVTT(−) tumor tissue (*R*^2^ = 0.0324, *P* = 0.04) and PVTT(+) tumor tissue (*R*^2^ = 0.5127, *P* = 0.006). (**C**) 14-3-3ζ expression in 10 PVTT(−) HCC tumor samples and 10 PVTT(+) HCC tumor samples were evaluated by western blot. **P < 0.01. (**D**) Relative 14-3-3ζ expression in normal liver tissues, PVTT(−) HCC tissues and PVTT(+) HCC tissues among 143 HCC samples. Representative IHC images are shown in (Left panel). **P < 0.01.

Since HIF-1α plays a crucial role in hypoxia [12], we sought to identify proteins interacting with HIF-1α under the assumption that these proteins will be potential drug targets to inhibit HIF-1α. We first screened proteins associated with HIF-1α using Scansite software (http://www.motifscan.mit.edu). 35 proteins were found to potentially interact with HIF-1α (Figure 2A, left panel and Supplementary Table 1). We next performed cDNA microarray analysis using paired HCC/normal tissues (*n* = 5) to profile the genes up-regulated in HCC. 1012 genes were found increased in tumor tissues compared with the matched nontumorous tissues (Figure 2A, left panel and Supplementary Table 2). Combined analysis of both results from bioinformatics and microarray analysis showed that only YWHAZ (also known as 14-3-3ζ) and PRKDC were potential HIF-1α binding proteins that were also increased in HCC (Figure 2A, left panel and Supplementary Figure 1).

14-3-3ζ was selected for further investigation due to its correlation with tumor metastasis and functions in regulating proteins stability [13]. Gene set enrichment analysis (GSEA) confirmed the positive correlation of 14-3-3ζ with cell migration and tumor invasiveness (Figure 2A, right panel). The previous IHC analysis using TMAs (Figure 1) showed a positive linear correlation between the expression of 14-3-3ζ and HIF-1α in primary tumor tissues, especially in PVTT(+) tumor tissues (Figure 2B), suggesting a potential relationship between the two proteins.

We then investigated 14-3-3ζ expression in HCC tissues using real-time PCR and western blots. Both 14-3-3ζ mRNA and protein were higher in tumor tissues (T) than that in nontumorous tissues (N) (Supplementary Figure 2A–2B). A similar result was observed in published data from Liver hepatocellular carcinoma (LIHC) in The Cancer Genome Atlas (TCGA) database (Supplementary Figure 2C). Then, the expression of 14-3-3ζ in HCC tumors from patients with (PVTT+, *n* = 10) or without PVTT (PVTT−, *n* = 10) was assessed by western blot to determine the relationship between 14-3-3ζ expression and PVTT status in HCC. 14-3-3ζ protein level was significantly higher in PVTT(+) tumors than in PVTT(−) tumors (Figure 2C). Similar results were also observed in IHC analysis (Figure 2D), further confirming the potential correlation between 14-3-3ζ overexpression and the PVTT formation.

### Hypoxia induces elevated expression of 14-3-3ζ in HCC cells

**Figure 3:**
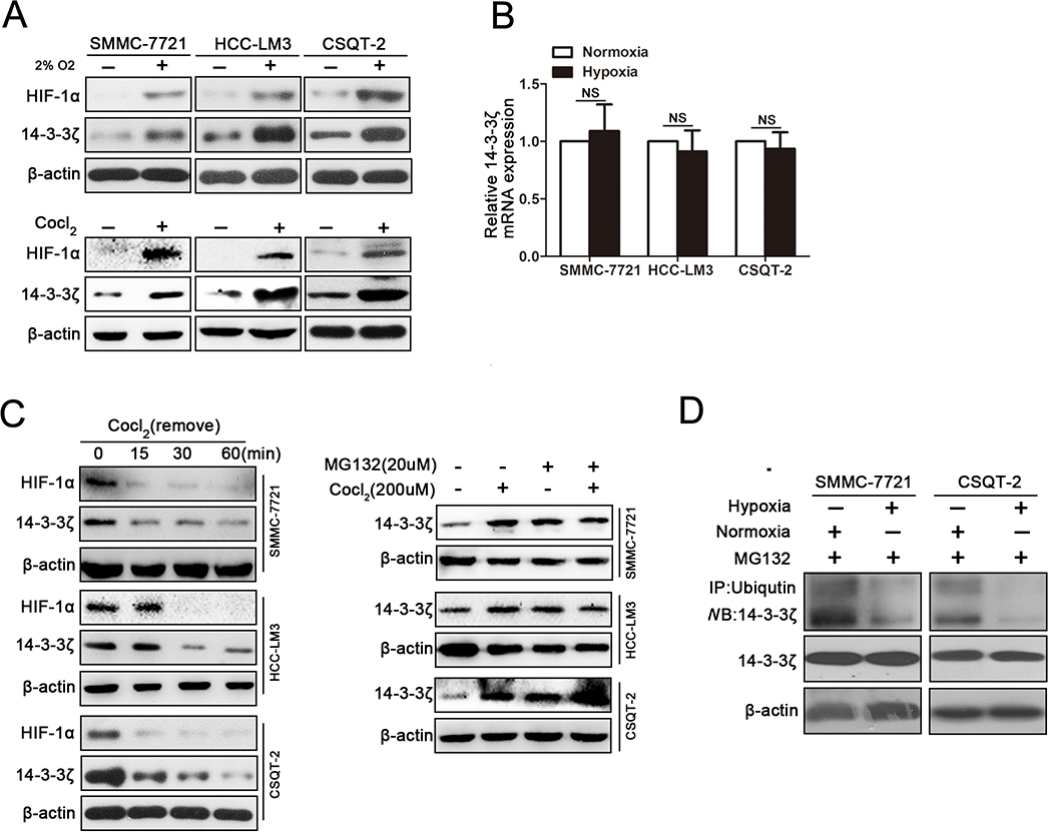
Hypoxia induces elevated expression of 14-3-3ζ in HCC cells (**A**) The expression of HIF-1α and 14-3-3ζ in tumor cell lines (SMMC-7721, HCC-LM3, CSQT-2) under normoxic/hypoxic conditions detected by western blot. (**B**) The mRNA levels of 14-3-3ζ in SMMC-7721, HCC-LM3 and CSQT-2 cells were determined by qRT-PCR. Data are mean ± SEM. and are representative of three independent experiments. NS: no significant, *P* > 0.05. (**C**) 14-3-3ζ protein in SMMC-7721, HCC-LM3 and CSQT-2 cells under different conditions (left panel, treated with CoCl2 18 hours and then incubation with normal fresh medium for the indicated times; right panel, incubated with CoCl2 or MG132). (**D**) The indicated cell lysates were prepared and immunoprecipitated using an agarose-conjugated anti-14-3-3ζ antibody. The immunoprecipitates and cell lysates were analyzed using anti-ubiquitin antibody by western blotting.

Because both intratumoral hypoxia status and 14-3-3ζ expression were associated with PVTT formation, we next asked whether hypoxia could induce 14-3-3ζ expression in HCC cells. When HCC cell lines were incubated under hypoxic conditions (2% O_2_), elevated 14-3-3ζ expression was observed in these cell lines (Figure 3A). Moreover, Cobalt chloride (CoCl_2_) treatment, which mimics hypoxic conditions *in vitro*, also elevated 14-3-3ζ expression in HCC cells (Figure 3A). Moreover, at different time points after CoCl2 exposure, 14-3-3ζ expression increased in parallel with hypoxia status (Supplementary Figure 3A), which was marked by the expression of HIF-1α.

To evaluate whether 14-3-3ζ is a target gene of HiF-1α, we next examined the expression of 14-3-3ζ mRNA in HCC cells under hypoxic conditions. Interestingly, 14-3-3ζ mRNA was not sensitive to CoCl_2_ treatment (Figure 3B) and HIF-1α interference did not affect 14-3-3ζ protein expression under hypoxic conditions (Supplementary Figure 3B), suggesting that hypoxia might increase 14-3-3ζ expression by affecting its protein stability. This hypothesis was confirmed by the finding that 14-3-3ζ protein declined immediately after CoCl_2_ was removed (Figure 3C, left panel). To further investigate whether degradation of 14-3-3ζ protein was inhibited by hypoxia, cell lines (SMMC-7721, HCC-LM3 and CSQT-2) treated with or without CoCl_2_ were further treated with a proteasome inhibitor, MG132 (20 μM). No change in 14-3-3ζ protein was observed in any of the cell lines between CoCl_2_-treated cells and control cells in the presence of MG132 (Figure 3C, right panel). Instead, ubiquitination of 14-3-3ζ was decreased under hypoxic conditions (Figure 3D). Taken together, these results indicated that hypoxia stabilizing 14-3-3ζ protein expression.

### 14-3-3ζ interacts with HIF-1α and enhances HIF-1α protein stability by recruiting HDAC4

**Figure 4:**
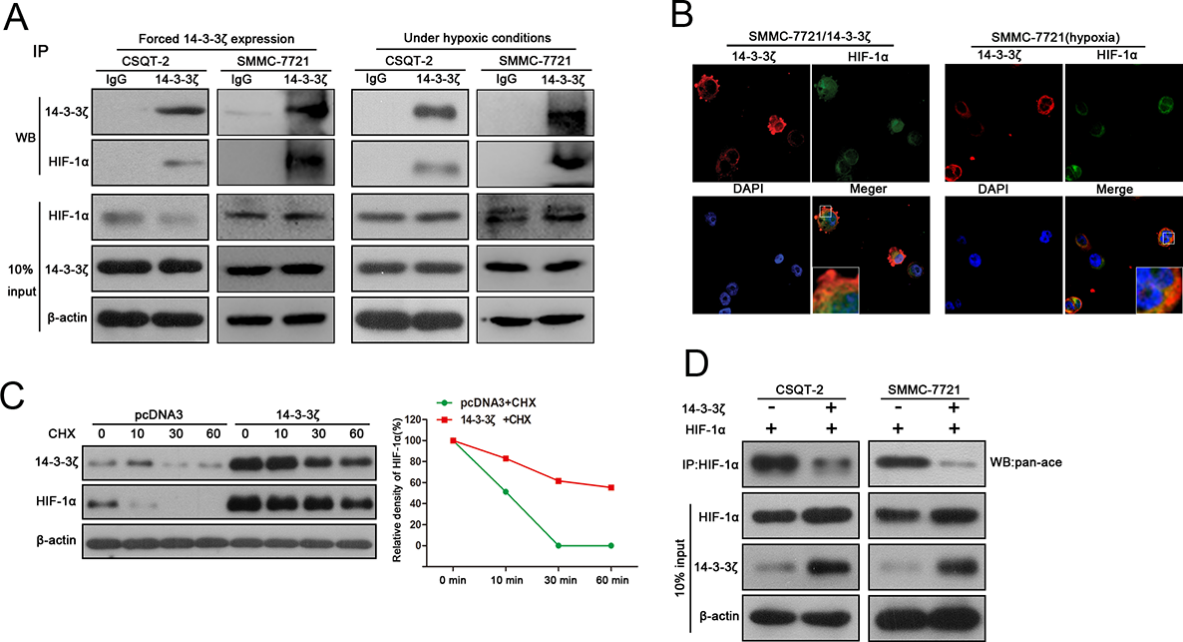
14-3-3ζ interacts with HIF-1α and enhances HIF-1α protein stability by recruiting HDAC4 (**A**) The interaction between HIF-1α and 14-3-3ζ was determined by co-IP assays in SMMC-7721 and CSQT-2 cells over-expressing 14-3-3ζ expression or treated with CoCl2. Cell-lysates was immunoprecipitated with anti-14-3-3ζ antibody, and then probed using anti-HIF-1α antibody. (**B**) Immunofluorescence assays were performed in SMMC-7721/14-3-3ζ cells and CoCl2-treatment in SMMC-7721 cells. The localization of 14-3-3ζ and HIF1α was detected using a Leica confocal microscope. (**C**) CSQT-2/pcDNA3 and CSQT-2/14-3-3ζ were treated with cycloheximide(CHX), and then the expression of HIF-1α and 14-3-3ζ were examined by western blot. (**D**) CSQT-2 and SMMC-7721 cells were co-transfected with HIF-1α and 14-3-3ζ, and then a co-IP assays were performed. Cell-lysates was immunoprecipitated with anti-HIF-1α antibody, and then probed using anti-pan-Ac antibody.

Since both 14-3-3ζ and HIF-1α are induced by hypoxia and were predicted to bind to each other by Scansite software, we investigated whether 14-3-3ζ regulates HIF-1α through protein-protein interactions. Tumor cells (CSQT-2, SMMC-7721) were transfected with plasmid expressing 14-3-3ζ and then lysed to perform a pull down assay using 14-3-3ζ antibody. Western blot detection of HIF-1α in the precipitates confirmed the interaction between 14-3-3ζ and HIF-1α (Figure 4A, left panel). The same experiments were also performed under hypoxic conditions and similar results were also observed (Figure 4A, right panel). For further verification, immunofluorescence was performed with SMMC-7721 cells expressing exogenous 14-3-3ζ or treated with CoCl_2_. Both 14-3-3ζ and HIF-1α proteins were co-localized in cytoplasm (Figure 4B), indicating the possibility for interactions between 14-3-3ζ and HIF-1α.

To investigate whether this interaction affects HIF-1α protein stability, CSQT-2 cells with or without ectopic 14-3-3ζ expression were treated with cycloheximide (CHX), an inhibitor of *de novo* protein synthesis. HIF-1α protein remains relatively stable up to 1 h in the presence of 14-3-3ζ and disappeared in the presence of pcDNA3 during this period (Figure 4C), indicating that 14-3-3ζ promotes the stability of HIF-1α. The stability of HIF-1α protein was enhanced by deacetylation through HDAC4 [14], which also physically interacts with 14-3-3 in mammalian cells [15]. Thus, to elucidate the underlying molecular mechanism of enhanced stability of HIF-1 α, we measured acetylated HIF-1α in tumor cells exogenously expressing 14-3-3ζ. We first validated the interaction between 14-3-3ζ and HDAC4 in CSQT-2 and SMMC-7721 cells under both normal and hypoxic conditions (Supplementary Figure 4A). When 14-3-3ζ was up-regulated in CSQT-2 and SMMC-7721 cells, acetylated HIF-1α was markedly decreased (Figure 4D), indicating that 14-3-3ζ may enhance HIF-1α protein stability by recruiting HDCA4 to inhibit HIF-1α acetylation. This result was corroborated by a reduction of HIF-1α protein in tumor cells with HDCA4 inhibition (Supplementary Figure 4B), as well as co-localization of HDAC4/HIF-1α, HDAC4/14-3-3 and HIF-1α/14-3-3 in SMMC-7721 cells under hypoxic conditions (Supplementary Figure 4C).

### 14-3-3ζ regulates the aggressive behavior of tumor cells by the HIF-1α/EMT signaling pathway under both hypoxic and normoxic conditions

**Figure 5:**
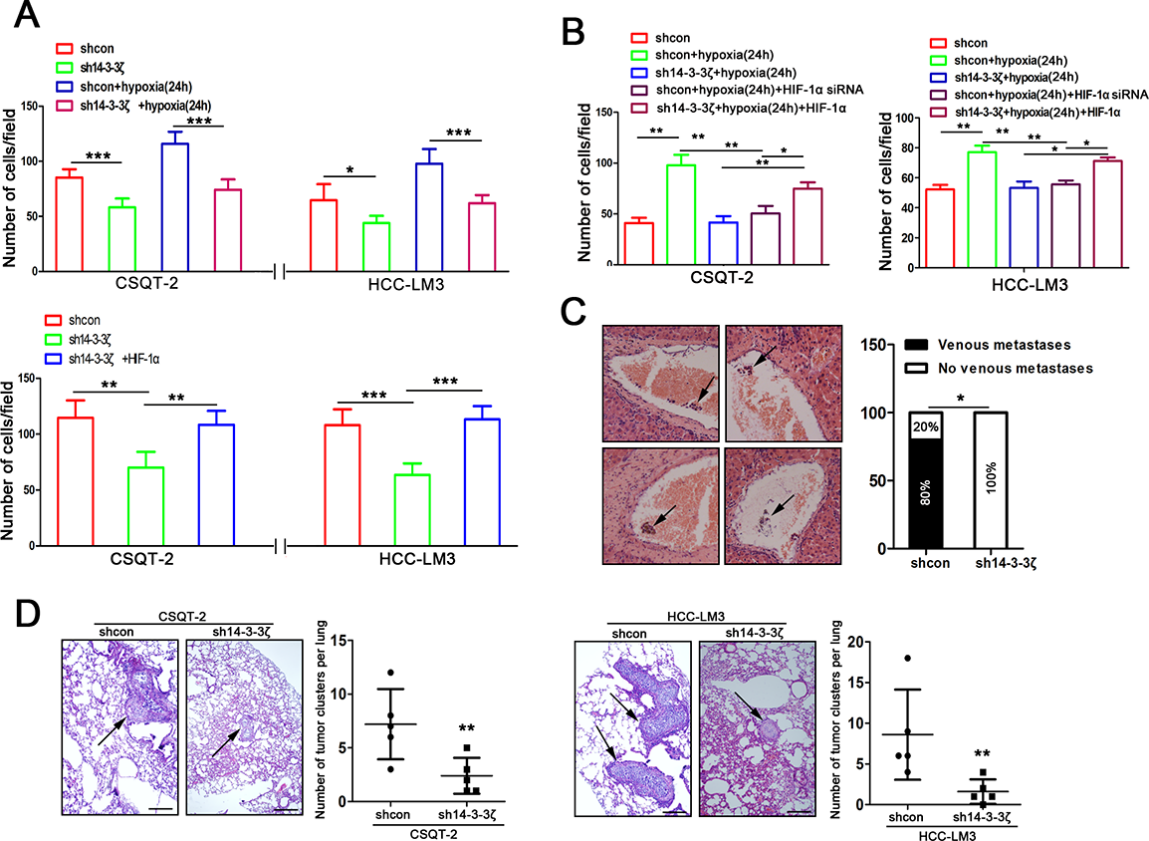
14-3-3ζ regulates the aggressive behavior of tumor cells by the HIF-1α/EMT signal pathway under hypoxic and normoxic conditions (**A**) The invasion ability of indicated cells with different treatments (upper panel, CSQT-2 shcon/sh14-3-3ζ cells and HCC-LM3 shcon/sh14-3-3ζ cells under normoxic/hypoxic conditions; lower panel, cells with or without ectopic HIF-1α expression under nomoxic conditions). Data are mean±SEM. and are representative of three independent experiments. **P < 0.01 and ***P < 0.001. (**B**) Under hypoxic conditions, invasion assays were performed for the indicated cells following treatment with or without modulated HIF-1α expression. Cell invasion was determined with crystal violet staining. Data are mean ± SEM and are representative of three independent experiments. *P < 0.05 and **P < 0.01. (**C**) PVTTs (indicated by arrows) were detected by liver HE staining in an orthotopic transplantation of nude mice which implanted with CSQT-2 cells (left panel). Incidences of PVTT was examined under the microscope (right panel). **P < 0.01. (**D**) Representative images of lung tissue sections from each group (hematoxylin and eosin stain (indicated by arrows, left panel); bars, 100 μm). The number of lung metastatic foci in each group (*n* = 5) of CSQT-2/shcon and CSQT-2/sh14-3-3ζ and HCC-LM3/shcon and HCC-LM3/sh14-3-3ζ xenografted mice was calculated microscopically 8 weeks after tail vein injection (right panel). **P < 0.01.

Having documented that hypoxia induced 14-3-3ζ expression regulated HIF-1α expression, we sought to elucidate the role of 14-3-3ζ in tumor cell behavior. Trans-well invasion assays were used to assess the potential invasion capacity of tumor cells with 14-3-3ζ modulation. 14-3-3ζ down-regulation reduced the invasion capacity of CSQT-2 and HCC-LM3 cells under both of normoxic and hypoxic conditions (Figure 5A and Supplementary Figure 5), which could be rescued by HIF-1α (Figure 5B). These results suggested that hypoxia-induced invasion of HCC cells is, at least in part, a result of activation of 14-3-3ζ/HIF-1α axis.

To verify the involvement of 14-3-3ζ in tumor metastasis *in vivo*, we performed orthotopic transplantation assays in nude mice. CSQT-2 cells were used because they are derived from PVTT and prone to extend to portal veins forming PVTT, and sh14-3-3ζ was used for knock-down as previously described [16]. The incidence of PVTT was lower in mice transplanted with CSQT-2/sh14-3-3ζ cells than in those transplanted with CSQT-2/shcon cells (Figure 5C). We also established a mouse model of PVTT by injecting HCC-CSQT-2/sh14-3-3ζ cells or HCC-CSQT-2/shcon cells into mice through the tail vein. Histopathological examination revealed fewer micrometastatic lesions in the lungs of mice injected with HCC-CSQT-2/sh14-3-3ζ cells than in those of control mice injected with HCC-CSQT-2/shcon cells (Figure 5D, left panel). Similar results were obtained using LM3 cells (Figure 5D, right panel). These results suggest that 14-3-3ζ down-regulation in HCC cells could inhibit metastases to lung and the formation of PVTT *in vivo*.

Epithelial–mesenchymal transition (EMT) favors cells disseminating from the site of a primary tumor and is a key process to initiate cell metastasis [17], thus, we asked whether 14-3-3ζ is involved in EMT of HCC cells. When 14-3-3ζ was exogenously expressed in SMMC-7721 cells (SMMC-7721/14-3-3ζ), some cells displayed a mesenchymal appearance, while the control cells (SMMC-7721) maintained the typical epithelial phenotype (Supplementary Figure 6A). Subsequent Immunofluorescence staining found vimentin expression in SMMC-7721 cells (SMMC-7721/14-3-3ζ) with a mesenchymal phenotype (Supplementary Figure 6B). Real-time PCR analysis found down-regulation of epithelial markers such as E-cadherin, ZO-1 and desmoplakin and up-regulation of mesenchymal markers such as snail, fibronectin and twist in 14-3-3ζ over-expressing SMMC-7721 cells (Supplementary Figure 6C). Reduced E-cadherin protein and increased vimentin and snail protein were also found in 14-3-3ζ over-expressing SMMC-7721 cells (Supplementary Figure 6D). When 14-3-3ζ was down-regulated in tumor cells, E-cadherin protein was up-regulated, and Vimentin and snail were down-regulated (Supplementary Figure 6E), suggesting a potential role for 14-3-3ζ in EMT of tumor cells.

In addition, we found that the up-regulation of E-cadherin and down-regulation of Vimentin and Snail induced by sh14-3-3ζ can be rescued by HIF-1α (Supplementary Figure 6F). Moreover, down-regulation of 14-3-3ζ inhibited hypoxia induced down-regulation of E-cadherin and up-regulation of Vimentin and Snail (Supplementary Figure 6G). The expressions of these proteins in 14-3-3ζ down-regulated tumor cells was rescued by ectopic HIF-1α expression under hypoxic conditions (Supplementary Figure 6G). These results suggest that 14-3-3ζ promotes HCC cells metastasis via the HIF-1α/EMT signaling pathway under hypoxic conditions.

### Combination of 14-3-3ζ and HIF-1α has a better prognostic value for HCC

**Figure 6:**
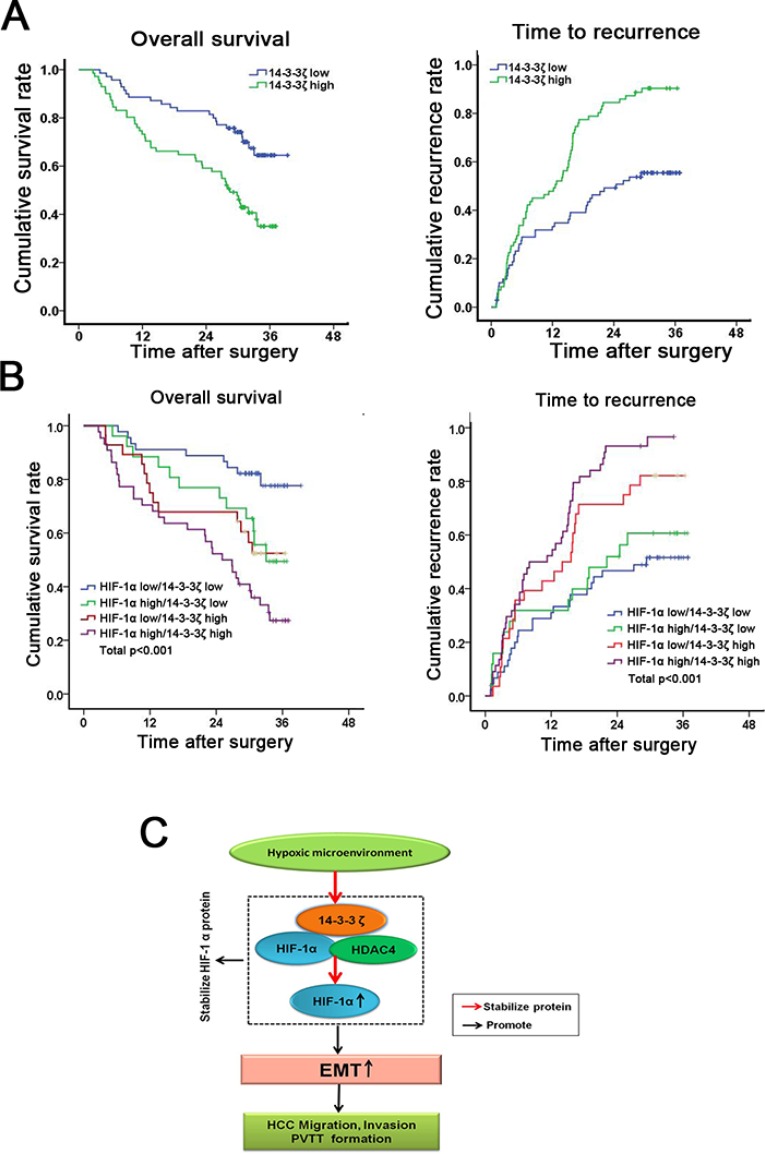
Combination of 14-3-3ζ and HIF-1α has a better prognostic value for HCC (**A**) The cumulative survival (left pane) and recurrence (right panel) rate were analyzed in 143 HCC patients. (**B**) The combination of 14-3-3ζ and HIF-1α increased the probability of a poor prognosis. (**C**) Schematic representation of the mechanism underlying hypoxia/14-3-3ζ/HIF-1α-facilitated HCC metastasis and PVTT formation.

Having documented the crucial role of 14-3-3ζ in HIF-1α regulation and PVTT formation, we next investigated the clinical correlation of 14-3-3ζ and HIF-1α expression in HCC tumors. Using IHC in paired HCC samples from 143 patients (Figure 1A and Figure 2D), patients were divided into a high group (*n* = 72) and a low group (*n* = 71) according to the intensity of HIF-1α or 14-3-3ζ staining. Both HIF-1α and14-3-3ζ expression were found to be correlated with tumor size (*P* < 0.05), microscopic vascular invasion (*P* < 0.05), PVTT (*P* < 0.05) and advanced tumor stage (*P* < 0.01) (Supplementary Tables 3 and 4, Supplementary Figure 7). Kaplan-Meier survival analysis was performed to assess the prognostic value of 14-3-3ζ expression. there were differences in both overall survival (OS; median OS times were 28 vs. > 33 months, respectively, difference > 5 months, *P* < 0.001) and time to recurrence (TTR; median TTR times were 12 vs. 24 months, respectively, difference = 12 months, *P* < 0.001) between the two groups (Figure 6A, Supplementary Table 5).

For patients whose tumors expressed above-average levels of HIF-1α (HIF-1α high), adverse outcomes were exacerbated (Supplementary Figure 8). moreover, the HCC patients with above-average levels of both HIF-1α and 14-3-3ζ (HIF-1α high/14-3-3ζ high) displayed even worse prognoses, indicating that a combination of these two parameters increases the prognostic value (Figure 6B). Thus, evaluation of both HIF-1α and 14-3-3ζ expression is a powerful predictor of poor prognosis in HCC, leading further support to a model of 14-3-3ζ increasing HIF-1α expression through enhancement of protein stability, resulting in an enhanced EMT followed by metastases of HCC cells and the formation of PVTT (Figure 6C).

## DISCUSSION

As an important microenvironmental factor of solid tumors, hypoxia plays an critical role in tumor metastasis including HCC. Although a positive relationship between intratumoral hypoxia (reflected by HIF-1α) and PVTT formation in HCC patients has been found in several clinical studies [18, 19], the mechanisms associated with hypoxia-induced PVTT formation are still obscure. In the current study we found that increased intratumoral hypoxia is strongly correlated with PVTT formation in HCC patients and the hypoxia/14-3-3ζ/HIF-1α signaling axis contributes to the PVTT development.

HIF-1α is a major factor regulating the cellular response to hypoxia. Aberrant expression of HIF-1α has been previously observed in almost every solid tumor, and HIF-1α has been found to promote growth and metastasis of tumors [8, 20]. Considering the central role of the interaction between HIF-1α and other proteins under hypoxic conditions [21], we investigated the novel target proteins of HIF-1α and investigated whether these target proteins contribute to hypoxia-induced PVTT formation. We showed that 14-3-3ζ, prevents acetylation of HIF-1α by recruiting HDAC4 protein, resulting in the increased stabilization of HIF-1α protein. Importantly, knocking-down of 14-3-3ζ attenuates hypoxia-induced invasion *in vitro* and inhibits PVTT formation as well as distant lung metastasis *in vivo*, indicating that 14-3-3ζ plays an important role in hypoxia-mediated metastasis and PVTT formation. Therefore, our results reveal for the first time that 14-3-3ζ functions as a positive regulator of HIF-1α, and the induction of HIF-1α by 14-3-3ζ augments the metastatic potential of tumor cells under hypoxic conditions.

14-3-3ζ was previously found to participate in the formation of breast cancer, non-small cell lung cancer (NSCLC), and head and neck squamous cell carcinomas (HNSCCs) [22–24]. Elevated 14-3-3ζ expression in these cancers promoted proliferation, inhibited apoptosis, and enhanced chemotherapy resistance in cancer cells. 14-3-3ζ was also increased in HCC, while 14-3-3ζ inhibition suppressed tumor cell proliferation and enhanced the anti-cancer effects of cis-diamminedichloridoplatium (CDDP) [25]. Additionally, 14-3-3ζ up-regulation was involved in tumor invasion and metastasis. By forming a complex with ErbB2 or αB-crystallin, 14-3-3ζ promoted tumor metastasis by inducing EMT of tumor cells. [26–28]. In accordance with these studies, in the present study we demonstrated elevated 14-3-3ζ expression in HCC (Supplementary Figure 2) and its role in HCC metastasis via EMT (Figure 5). Moreover, we found that 14-3-3ζ stabilized HIF-1α protein through preventing its acetylation via recruiting HDAC4 and then induced EMT of HCC cells, suggesting a novel pathway involving 14-3-3ζ. Aberrant expression of 14-3-3ζ, previously observed in several cancers, is largely a result of genomic alterations and post-transcriptional modulation of stability [13, 29]. Our results also showed that both 14-3-3ζ mRNA and protein are overexpressed in HCC tissues (Figure 2) and hypoxia increases 14-3-3ζ protein by inhibiting the proteasome-mediated degradation, while 14-3-3ζ mRNA was not affected by hypoxia (Figure 3). These results indicated that multiple mechanisms contribute to the aberrant expression of 14-3-3ζ. We speculated that genomic alterations increased 14-3-3ζ mRNA levels and that the hypoxic microenvironment further stabilizes 14-3-3ζ proteins, resulting in 14-3-3ζ overexpression in tumor tissues.

Although we have demonstrated that the hypoxia/14-3-3ζ/HIF-1α pathway plays an important role in HCC metastasis and PVTT formation, our study also raises several critical questions. Previous studies showed inflammatory cytokines, including TGF-β and IL-6, contribute to the PVTT formation [7, 30]. Hypoxia may also be involved in increasing TGF-β and IL-6 in tumor cells [31, 32]. In addition, several inflammatory cytokines, including TNF-α, IL-1, IL-6, TGF-β and IL-18, have been documented to increase HIF-1α expression or transcriptional activation in tumor cells [33–36]. Therefore, 14-3-3ζ may be involved in hypoxia-induced inflammatory cytokine expression, which also promotes PVTT formation in HCC patients. Additionally, our previous research found that non-coding RNAs such as miRNAs also involved in PVTT development [37]. Whether or not these non-coding RNAs also interact with the hypoxia/14-3-3ζ/HIF-1α pathway to promote PVTT development is also unclear. A comprehensive investigation for the synergistic actions of a hypoxic microenvironment, inflammatory responses, and non-coding RNAs will be helpful for revealing the full mechanism underlying the PVTT formation [36]. Moreover, in addition to 14-3-3ζ, several other isoforms of 14-3-3 such as 14-3-3β and 14-3-3ε have also been shown to contribute to cell migration and EMT of HCC [38, 39]. These other 14-3-3 isoforms are also overexpressed and associated with high metastatic risk and poorer survival rates of HCC [38–42]. However, whether or not these proteins are involved in PVTT formation and are regulated by the same mechanism in HCC remains unclear.

In conclusion, we found that hypoxia up-regulates 14-3-3ζ expression in HCC by increasing its protein stability through a ubiquitin pathway. 14-3-3ζ promotes hypoxia-induced HCC invasion *in vitro* and PVTT formation *in vivo* by stabilizing HIF-1α protein. These findings reveal a novel mechanism underlying PVTT formation by the hypoxia/14-3-3ζ/HIF-1α pathway, which may contribute to the development of new therapeutics for HCC patients.

## MATERIALS AND METHODS

### Patients and samples

This study was approved by the Ethics Committee of Eastern Hepatobiliary Surgery Hospital and all participants provided written consent. The criteria for inclusion and exclusion was in accordance with previous reports [16]. According to these criteria, 144 HCC patients (collected from June 2009 to May 2010, Supplementary Table 6), who underwent curative resection in the Eastern Hepatobiliary Surgery Hospital (Shanghai, China) were randomly retrieved to construct a tissue microarray (TMA) as previously described [43]. Each patient was followed until September 2012 with the longest follow-up up to 39 months. The time of the surgery was used to calculate the time to a given event. Overall survival (OS) and time to recurrence (TTR) were defined as previously described [44].

In addition, 76 pairs of tumor and nontumorous samples (collected from October 2011 to July 2012), 20 PVTT(+) and 20 PVTT(−) primary tumor tissues (collected from March 2014 to September 2014) were used to perform real-time PCR and western blot.

### Cell lines and cell culture

The liver cancer cell line SMMC-7721 was purchased from the Cell Bank of the Chinese Academy of Sciences (Shanghai, China). The MHCC-LM3 liver cancer cell lines was provided by Professor Wei-zhong Wu (Zhong Shan Hospital, Fu Dan University, Shanghai, China). The PVTT-originated cell line CSQT-2 was generated in our laboratory [16]. Cells were maintained at 37°C in a humidified incubator containing 5% CO_2_ in Dulbecco's Modified Eagle's Medium (Gibco-BRL, CO.LTD, USA), supplemented with 10% heat-inactivated fetal bovine serum (Gibco-BRL, CO.LTD, USA) and passaged every 3 days to maintain logarithmic growth. To mimic hypoxic conditions, the cells were incubated in the presence of 200 μM cobalt chloride (CoCl_2_, St. Louis, MO USA).

### Microarray analysis

Total RNA was isolated from 5 paired HCC tissues (tumor tissues and matched nontumorous tissues using Trizol reagent (Invitrogen) and was sent to Gminix

Co., Ltd (Shanghai) for cDNA microarray analysis as previously described [45].

### Bioinformatics analysis

The liver hepatocellular carcinoma (LIHC) dataset consisting of 371 HCC tumor tissues and 50 non-tumorous tissues was downloaded from TCGA website (https://tcga-data.nci.nih.gov/tcga/) following approval of this project by the consortium. To probe the YWHAZ (14-3-3ζ) gene-associated pathways in an unbiased basis way, we performed gene set enrichment analysis (GSEA) using GSEA version 2.0 from the Broad Institute at MIT. Expression of the gene was used as a phenotype label, and “Metric for ranking genes” was set to Pearson Correlation.

### Plasmid construction and stable transfection

The pcDNA3 14-3-3ζ plasmid (ID: 9002) was obtained from Addgene (Cambridge, MA, American). The PLKo-sh14-3-3ζ plasmid was obtained by cloning14-3-3ζ-small hairpin RNA into the pLKO vector (Invitrogen, St. Louis, MO, USA). All of the constructs were confirmed using DNA sequencing and western blot analyses. Transfections were performed using the Lipofectamine 2000 Kit (Invitrogen, Carlsbad, CA, USA) according to the manufacturer's recommended instructions. Stable 14-3-3ζ and sh14-3-3ζ expression was established in hepatoma cells using Geneticin (G418) (Sigma, USA) selection.

### RNA interference

The HIF-1α siRNA (h) sequences were as follows: siRNA1 (sense, 5′–3′: CCACCACUGAUGAA UUAAATT, and antisense, 5′–3′: UUUAAUUCAUCA GUGGUGGTT) and siRNA2 (sense, 5′–3′:GCUGGAG ACACAAUCAUAUTT, and antisense, 5′–3′: AUAUGA UUGUGUCUCCAGCTT). The negative control siRNA sequences were as follows: sense, 5′–3′, UUCUCCGAAC GUGUCA CGUT, and antisense, 5′–3′, ACGUGACAC GUUCGGAGAATT. All of these siRNAs were purchased from GenePharma Company (Shanghai, China). For the transient transfections, cells were transfected with Lipofectamine 2000 according to the manufacturer's recommended instructions.

### Reverse transcriptase-polymerase chain reaction (RT-PCR) and quantitative RT-PCR

Total RNA was isolated from the cell lines and clinical samples using TRIzol reagent according to the manufacturer's recommended protocol (Invitrogen, Carlsbad, CA). RT-PCR performed as described previously with specific primers [16]. Quantitative RT-PCR was performed using SYBR Premix Ex Taq^™^ (TaKaRa BIOTECHNOLOGY CO., LTD, Dalian, China) and the StepOnePlus Real-Time PCR System (Applied Biosystems, Foster City, CA). Gene expression was calculated relative to the expression of β-actin in tumor cell lines or clinical samples using the 2^−ΔΔCt^ method. The primers used were provided in the Supplementary Table 7.

### Western blot analyses

Western blot analyses were performed as previously described [46]. The total soluble proteins (40 μg) were used and β-actin was used as an internal control. The antibodies used are listed in the Supplementary Table 8.

### Immunohistochemical analyses

Paraffin-embedded tissue sections or tissue microarray slides were analyzed using immunohistochemistry (IHC) as previously described [47]. The antibodies that were used for IHC are listed in Supplementary Table 8. Slides were scanned with an Aperio ScanScope GL. Aperio ImageScope software (Aperio Technologies, Vista, CA) was then used to assess the staining of the scanned images based on the percentage of positively stained cells and the staining intensity. Scores were then generated by software.

### Evaluation of PI3K activity and NF-κB activity

SMMC-7721/14-3-3ζ, MHCC/sh14-3-3ζ or control cells were used to examined the PI3K activity and NF-κB activity as described previously [43, 48].

### *In vitro* cell behavior assays

CSQT-2/14-3-3ζ, MHCC/sh14-3-3ζ, SMMC-7721/14-3-3ζ or control cells were subjected to invasionassays using Millicell inserts (Millipore, Billerica, M A, USA), which had been coated with Matrigel (BD Biosciences, Sparks, MD, USA). Cells (5.0 × 10^4^) were plated onto the upper chambers. After 24 hours, the non-invasive cells on the upper chambers were removed using a cotton swab and the cells that had invaded into the underside of the membrane were stained using crystal violet staining. Cell numbers were plotted as the average number of invaded cells from 5 random microscopic fields.

### *In vivo* metastasis assays

CSQT-2/sh14-3-3ζ, MHCC-LM3/sh14-3-3ζ or control cells were subjected to metastasis assays *in vivo*. Twenty 6-week-old male nude mice were used in metastasis assays. 2 × 10^6^ cells were used to inject into tail vein of each nude mice. The mice were sacrificed at 8 weeks post-injection and examined microscopically using H and E staining for the development of lung metastatic foci. All of the mice were maintained in a pathogen-free facility and used in accordance with the institutional guidelines for animal care.

### Orthotopic transplantation

CSQT-2/sh14-3-3ζ and control cells were used in orthotopic transplantation, which were performed as previously described [16].

### Immunoprecipitation

Immunoprecipitation was performed on total protein extracts from cells by following a routine protocol [15]. The antibodies that were used are listed in Supplementary Table 8.

### Immunofluorescence assay

SMMC-7721/14-3-3ζ and control cells were used to reveal the location of vimentin. SMMC-7721/14-3-3ζ and SMMC-7721 cells that had been incubated with CoCl_2_ were used to reveal the location of 14-3-3ζ and HIF-1α. Monoclonal mouse anti-human vimentin (1:100; eBioscience), polyclonal rabbit anti-human 14-3-3ζ (1:100; Abcam) and polyclonal rabbit anti-human HIF-1α antibodies (1:100; Abcam) were used for fluorescence staining. DAPI was used to stain the nuclei. The coverslips were imaged using a Leica confocal microscope (Leica, Wetzlar, Germany). The data were processed using Adobe Photoshop 7.0 software.

### Statistical analyses

Statistical analyses were performed using SAS 9.1.3 software (SAS Institute Inc, Cary, NC). Student's *t*-tests were used to compare two groups, unless otherwise indicated (χ^2^ test). Quantitative variables were analyzed using student's *t*-test or Pearson's correlation test. Kaplan-Meier and log-rank analyses were used to assess patient survival between subgroups. The data were presented as the means ± SEM, unless otherwise indicated. *P* < 0.05 was considered to be statistically significant.

## SUPPLEMENTARY MATERIALS FIGURES AND TABLES


