# Supplementary material for: 14-3-3ζ promotes hepatocellular carcinoma venous metastasis by modulating hypoxia-inducible factor-1α
Source: Oncotarget. 2016 Feb 19;7(13):15854–67. doi: 10.18632/oncotarget.7493 (PMC4941282; doi:10.18632/oncotarget.7493)
Supplement: Supplementary file 1 [file oncotarget-07-15854-s001.pdf]

14-3-3ζ promotes hepatocellular carcinoma venous metastasis by modulating hypoxia-inducible factor-1α

Supplementary Materials

Supplementary Table S1: The predicted target proteins of HIF-1α

Gene symbol	Motif group	Number of predicted binding site
YWHAZ	Phosphoserine/threonine binding group (pST_bind)	3
EGFR	Tyrosine kinase group (Y_kin)	1
PDGFRB	Tyrosine kinase group (Y_kin)	1
ITK	Tyrosine kinase group (Y_kin)	3
FGR	Tyrosine kinase group (Y_kin)	1
PIK3R1	Src homology 2 group (SH2)	1
CRK	Src homology 2 group (SH2)	1
FGR	Src homology 2 group (SH2)	1
LCK	Src homology 2 group (SH2)	1
SRC1	Src homology 2 group (SH2)	1
SHC1	Src homology 2 group (SH2)	1
ABL1	Src homology 2 group (SH2)	3
FYN	Src homology 2 group (SH2)	1
NCK1	Src homology 2 group (SH2)	2
NCK1	Src homology 3 group (SH3)	1
PRKCA	Basophilic serine/threonine kinase group (Baso_ST_kin)	7
PRKCD	Basophilic serine/threonine kinase group (Baso_ST_kin)	7
PRKCE	Basophilic serine/threonine kinase group (Baso_ST_kin)	5
PRKCM	Basophilic serine/threonine kinase group (Baso_ST_kin)	4
PRKCZ	Basophilic serine/threonine kinase group (Baso_ST_kin)	5
PRKACG	Basophilic serine/threonine kinase group (Baso_ST_kin)	3
CAMK2G	Basophilic serine/threonine kinase group (Baso_ST_kin)	5
AKT1	Basophilic serine/threonine kinase group (Baso_ST_kin)	1
PRKDC	DNA damage kinase group (DNA_dam_kin)	6
ATM	DNA damage kinase group (DNA_dam_kin)	4
CSNK1G2	Acidophilic serine/threonine kinase group (Acid_ST_kin)	12
CSNK2B	Acidophilic serine/threonine kinase group (Acid_ST_kin)	3
GSK3A	Acidophilic serine/threonine kinase group (Acid_ST_kin)	4
GSK3B	Acidophilic serine/threonine kinase group (Acid_ST_kin)	3
ATM	Acidophilic serine/threonine kinase group (Acid_ST_kin)	1
AMPH	Acidophilic serine/threonine kinase group (Acid_ST_kin)	1
CDC2	Proline-dependent serine/threonine kinase group (Pro_ST_kin)	3
CDC5	Proline-dependent serine/threonine kinase group (Pro_ST_kin)	5
MAPK3	Proline-dependent serine/threonine kinase group (Pro_ST_kin)	3
MAPK14	Proline-dependent serine/threonine kinase group (Pro_ST_kin)	1
SORBS1	Proline-dependent serine/threonine kinase group (Pro_ST_kin)	1
MAPK1	Kinase binding site group (Kin_bind)	1

Note: The higher sensitivity level has been chose to scan motifs.

Supplementary Table S2: Part of genes overexpressed in tumor tissues

Gene Symbol	Gene Title	RefSeq Transcript ID	*p*-value	Fold Change
PRKDC	protein kinase, DNA-activated, catalytic polypeptide	NM_001081640 / NM_006904	< 0.01	2.08765
GTF2H2	general transcription factor IIH, polypeptide 2	NM_001042490 / NM_001098728 / NM_001515 /NR_033417	< 0.05	2.08771
RAB7L1	RAB7, member RAS oncogene family-like 1	NM_001135662 / NM_001135663 / NM_001135664 / NM_003929	< 0.01	2.08898
CPSF2	cleavage and polyadenylation specific factor 2	NM_017437	< 0.01	2.09016
CCT5	chaperonin containing TCP1, subunit 5 (epsilon)	NM_012073	< 0.001	2.09066
GPATCH4	G patch domain containing 4	NM_015590 / NM_182679	< 0.01	2.09067
CLIC5	chloride intracellular channel 5	NM_001114086 / NM_016929	< 0.05	2.09071
PSME3	Proteasome macropain activator subunit 3	NM_005789 / NM_176863	< 0.01	2.09181
ALS2CR4	amyotrophic lateral sclerosis 2 (juvenile) chromosome region, candidate 4	NM_001044385 / NM_152388	< 0.05	2.09188
LASP1	LIM and SH3 protein 1	NM_006148	< 0.001	2.09334
PDZD11	PDZ domain containing 11	NM_016484	< 0.01	2.09346
RBM18	RNA binding motif protein 18	NM_033117 / NR_027125 / NR_027126	< 0.01	2.09371
ITGB3BP	integrin beta 3 binding protein (beta3-endonexin)	NM_014288	< 0.01	2.09391
STAT1	signal transducer and activator of transcription 1	NM_007315 / NM_139266	< 0.05	2.09468
PHTF2	putative homeodomain transcription factor 2	NM_001127357 / NM_001127358 / NM_001127359 / NM_001127360 / NM_020432	< 0.001	2.09511
RRP15	ribosomal RNA processing 15 homolog (S. cerevisiae)	NM_016052	< 0.01	2.09609
THEM4	thioesterase superfamily member 4	NM_053055	< 0.01	2.09631
ADSL	adenylosuccinate lyase	NM_000026 / NM_001123378	< 0.05	2.09639
NRAS	neuroblastoma RAS viral (v-ras) oncogene homolog	NM_002524	< 0.001	2.09641
CCDC99	coiled-coil domain containing 99	NM_017785	< 0.01	2.09716
RAB23	RAB23, member RAS oncogene family	NM_016277 / NM_183227	< 0.01	2.09717
PHPT1	phosphohistidine phosphatase 1	NM_001135861 / NM_014172	< 0.05	2.09784
ZNF354A	zinc finger protein 354A	NM_005649	< 0.001	2.09809

ACTR3	ARP3 actin-related protein 3 homolog (yeast)	NM_005721	< 0.001	2.09864
FAM50A	family with sequence similarity 50, member A	NM_004699	< 0.001	2.09957
GTPBP4	GTP binding protein 4	NM_012341	< 0.001	2.10004
ITGB3BP	integrin beta 3 binding protein (beta3-endonexin)	NM_014288	< 0.01	2.10036
PAM	peptidylglycine alpha-amidating monooxygenase	NM_000919 / NM_001177306 / NM_138766 / NM_138821 / NM_138822 / NR_033440	< 0.05	2.10044
SPAG5	sperm associated antigen 5	NM_006461	< 0.05	2.1005
STRBP	spermatid perinuclear RNA binding protein	NM_001171137 / NM_018387 / NR_033234	< 0.05	2.10195
ACLY	ATP citrate lyase	NM_001096 / NM_198830	< 0.01	2.10325
SRM	spermidine synthase	NM_003132	< 0.05	2.10335
LASP1	LIM and SH3 protein 1	NM_006148	< 0.001	2.10365
SAP30	Sin3A-associated protein	NM_003864	< 0.001	2.10463
DBF4	DBF4 homolog	NM_006716	< 0.01	2.10497
C8orf33	chromosome 8 open reading frame 33	NM_023080	< 0.05	2.10535
MAP4K4	mitogen-activated protein kinase kinase kinase kinase 4	NM_004834 / NM_145686 / NM_145687	< 0.01	2.10571
PAK1IP1	PAK1 interacting protein 1	NM_017906	< 0.01	2.10706
ALS2CR4	amyotrophic lateral sclerosis 2 (juvenile) chromosome region, candidate 4	NM_001044385 / NM_152388	< 0.05	2.10776
ASAP1	ArfGAP with SH3 domain, ankyrin repeat and PH domain 1	NM_018482	< 0.05	2.10889
OLA1	Obg-like ATPase 1	NM_001011708 / NM_013341	< 0.01	2.10946
ZNF43	zinc finger protein 43	NM_003423	< 0.05	2.10946
BLVRA	biliverdin reductase A	NM_000712	< 0.01	2.10952
BTG3	BTG family, member 3	NM_001130914 / NM_006806	< 0.001	2.11017
DR1	down-regulator of transcription 1, TBP-binding (negative cofactor 2)	NM_001938	< 0.01	2.11052
MANF	mesencephalic astrocyte-derived neurotrophic factor	NM_006010	< 0.01	2.11065
CNOT6	CCR4-NOT transcription complex, subunit 6	NM_015455	< 0.001	2.11138
MARS	methionyl-tRNA synthetase	NM_004990	< 0.001	2.11224
PPHLN1	periphilin 1	NM_001143787 / NM_001143788 / NM_001143789 / NM_016488 / NM_201438 / NM_20143	< 0.001	2.11357
PFDN4	prefoldin subunit 4	NM_002623	< 0.01	2.11374

PARP12	poly (ADP-ribose) polymerase family, member 12	NM_022750	< 0.001	2.11798
RRAGD	Ras-related GTP binding D	NM_021244	< 0.05	2.11846
TSPAN17	tetraspanin 17	NM_001006616 / NM_012171 / NM_130465	< 0.05	2.12102
MFSD6	major facilitator superfamily domain containing 6	NM_017694	< 0.05	2.12196
PPHLN1	periphilin 1	NM_001143787 / NM_001143788 / NM_001143789 / NM_016488 / NM_201438 / NM_20143	< 0.001	2.1229
MKI67	antigen identified by monoclonal antibody Ki-67	NM_001145966 / NM_002417	< 0.01	2.12295
ATP6V1C1	ATPase, H+ transporting, lysosomal , V1 subunit C1	NM_001695	< 0.05	2.12343
TDP1	tyrosyl-DNA phosphodiesterase 1	NM_001008744 / NM_018319	< 0.05	2.12485
H2AFY	H2A histone family, member Y	NM_001040158 / NM_004893 / NM_138609 / NM_138610	< 0.001	2.12565
PRKD3	protein kinase D3	NM_005813	< 0.001	2.12581
MRPS23	mitochondrial ribosomal protein S23	NM_016070	< 0.05	2.12848
SEPT8	septin 8	NM_001098811 / NM_001098812 / NM_001098813 / NM_015146	< 0.01	2.12931
DRAM2	DNA-damage regulated autophagy modulator 2	NM_178454	< 0.001	2.1297
DLAT	dihydrolipoamide S-acetyltransferase	NM_001931	< 0.05	2.13032
LPL	lipoprotein lipase	NM_000237	< 0.001	2.13065
IMPAD1	inositol monophosphatase domain containing 1	NM_017813	< 0.01	2.13114
LMNA	lamin A/C	NM_005572 / NM_170707/ NM_170708	< 0.01	2.13124
YWHAH	Tyrosine 3-monooxygenase/ tryptophan 5-monooxygenase activation protein, eta polypeptide	NM_003405	< 0.01	2.13276
SPON2	spondin 2, extracellular matrix protein	NM_001128325 / NM_012445	< 0.05	2.13529
SNRPD1	small nuclear ribonucleoprotein D1 polypeptide 16kDa	NM_006938	< 0.05	2.13545
HEATR1	HEAT repeat containing 1	NM_018072	< 0.01	2.13632
SEPT6	septin 6	NM_015129 / NM_145799 / NM_145800 / NM_145802	< 0.05	2.1373
ARMCX1	armadillo repeat containing, X-linked 1	NM_016608	< 0.05	2.13903

NIPA1	non imprinted in Prader-Willi/ Angelman syndrome 1	NM_001142275 / NM_144599	< 0.05	2.14062
MCM7	minichromosome maintenance complex component 7	NM_005916 / NM_182776	< 0.01	2.14085
GSR	glutathione reductase	NM_000637 / NM_001195102 / NM_001195103 / NM_001195104	< 0.05	2.14135
SLC36A1	solute carrier family 36 (proton/ amino acid symporter), member 1	NM_078483	< 0.01	2.14154
CLIC1	chloride intracellular channel 1	NM_001288	< 0.01	2.14156
TMEM184B	transmembrane protein 184B	NM_001195071 / NM_001195072 / NM_012264	< 0.001	2.14164
FBXO5	F-box protein 5	NM_001142522 / NM_012177	< 0.01	2.14204
RNF2	ring finger protein 2	NM_007212	< 0.001	2.14214
SLC2A5	solute carrier family 2 (facilitated glucose/fructose transporter), member 5	NM_001135585 / NM_003039 / NR_024180	< 0.05	2.14323
NPC1	Niemann-Pick disease, type C1	NM_000271	< 0.001	2.14339
AACS	acetoacetyl-CoA synthetase	NM_023928	< 0.001	2.14344
NSMAF	neutral sphingomyelinase (N-SMase) activation associated factor	NM_001144772 / NM_003580	< 0.01	2.1438
NMB	neuromedin B	NM_021077 / NM_205858	< 0.01	2.14399
GBP7	guanylate binding protein 7	NM_207398	< 0.05	2.14422
PPARG	peroxisome proliferator-activated receptor gamma	NM_005037 / NM_015869 / NM_138711 / NM_138712	< 0.05	2.14497
CDC73	cell division cycle 73, Paf1/ RNA polymerase II complex component, homolog (S. cerevisia	NM_024529	< 0.01	2.14527
DOCK11	dedicator of cytokinesis 11	NM_144658	< 0.05	2.14553
BAK1	BCL2-antagonist/killer 1	NM_001188	< 0.001	2.14561
ZBED4	zinc finger, BED-type containing 4	NM_014838	< 0.01	2.14625
TAGLN2	transgelin 2	NM_003564	< 0.001	2.14677
ILF2	interleukin enhancer binding factor 2	NM_004515	< 0.001	2.14894
C1orf112	chromosome 1 open reading frame 112	NM_018186	< 0.01	2.1492
C5orf28	chromosome 5 open reading frame 28	NM_022483	< 0.05	2.14942
TP53BP2	tumor protein p53 binding protein, 2	NM_001031685 / NM_005426	< 0.05	2.15015
UTP15	UTP15, U3 small nucleolar ribonucleoprotein, homolog (S. cerevisiae)	NM_032175	< 0.05	2.15197

MAP1B	microtubule-associated protein 1B	NM_005909	< 0.05	2.15237
CCT3	chaperonin containing TCP1, subunit 3 (gamma)	NM_001008800 / NM_005998 / NR_036564 / NR_036565	< 0.01	2.1524
SMC2	structural maintenance of chromosomes 2	NM_001042550 / NM_001042551/ NM_006444	< 0.001	2.15496
R3HDM1	R3H domain containing 1	NM_015361	< 0.001	2.15776
PNO1	partner of NOB1 homolog (S. cerevisiae)	NM_020143	< 0.001	2.15789
COMMD8	COMM domain containing 8	NM_017845	< 0.001	2.15797
ENPP2	ectonucleotide pyrophosphatase/ phosphodiesterase 2	NM_001040092 / NM_001130863 / NM_006209	< 0.05	2.15872
FOXM1	forkhead box M1	NM_021953 / NM_202002 / NM_202003	< 0.01	2.1597
ITGAV	integrin, alpha V (vitronectin receptor, alpha polypeptide, antigen CD51)	NM_001144999 / NM_001145000 / NM_002210	< 0.05	2.16003
UACA	uveal autoantigen with coiled-coil domains and ankyrin repeats	NM_001008224 / NM_018003	< 0.01	2.16018
C7orf31	chromosome 7 open reading frame 31	NM_138811	< 0.05	2.1624
WSB2	WD repeat and SOCS box-containing 2	NM_018639	< 0.05	2.1624
ZNF148	zinc finger protein 148	NM_021964	< 0.05	2.16248
GDAP1	ganglioside-induced differentiation-associated protein 1	NM_001040875 / NM_018972	< 0.05	2.16269
GLIS2	GLIS family zinc finger 2	NM_032575	< 0.01	2.1633
GPD2	glycerol-3-phosphate dehydrogenase 2 (mitochondrial)	NM_000408 / NM_001083112	< 0.05	2.16519
UHRF1	ubiquitin-like with PHD and ring finger domains 1	NM_001048201 / NM_013282	< 0.05	2.16742
WBP5	WW domain binding protein 5	NM_001006612 / NM_001006613 / NM_001006614 / NM_016303	< 0.05	2.16754
GALNT10	UDP-N-acetyl-alpha-D-galactosamine:polypeptide N-acetylgalactosaminyl-transferase 10	NM_198321	< 0.001	2.16756
CDC25B	cell division cycle 25 homolog B	NM_004358 / NM_021872 / NM_021873	< 0.001	2.16977
CORO1C	coronin, actin binding protein, 1C	NM_014325	< 0.01	2.17093
WSB2	WD repeat and SOCS box-containing 2	NM_018639	< 0.01	2.17103

ZWILCH	Zwilch, kinetochore associated, homolog	NM_017975 / NR_003105	< 0.001	2.17118
ENO1	enolase 1, (alpha)	NM_001428	< 0.05	2.17198
ALS2CR4	amyotrophic lateral sclerosis 2 (juvenile) chromosome region, candidate 4	NM_001044385 / NM_152388	< 0.05	2.173
MARS	methionyl-tRNA synthetase	NM_004990	< 0.001	2.17306
RQCD1	RCD1 required for cell differentiation1 homolog	NM_005444	< 0.01	2.17339
ACTR3	ARP3 actin-related protein 3 homolog	NM_005721	< 0.001	2.17403
TCEB1	transcription elongation factor B (SIII), polypeptide 1	NM_005648	< 0.05	2.17518
ANP32E	acidic (leucine-rich) nuclear phosphoprotein 32 family, member E	NM_001136478 / NM_001136479 / NM_030920	< 0.001	2.17529
ATP6V1C1	ATPase, H+ transporting, lysosomal, V1 subunit C1	NM_001695	< 0.05	2.17768
TMEM65	transmembrane protein 65	NM_194291	< 0.05	2.17816
CLIC1	chloride intracellular channel 1	NM_001288	< 0.01	2.17906
MKI67	antigen identified by monoclonal antibody Ki-67	NM_001145966 / NM_002417	< 0.01	2.17941
C18orf54	chromosome 18 open reading frame 54	NM_173529	< 0.01	2.17982
H2AFV	H2A histone family, member V	NM_012412 / NM_138635 / NM_201436 / NM_201516 / NM_201517	< 0.001	2.18049
COL11A1	collagen, type XI, alpha 1	NM_001190709 / NM_001854 / NM_080629 / NM_080630	< 0.05	2.18085
FTH1	ferritin, heavy polypeptide 1	NM_002032	< 0.05	2.18427
RRAGC	Ras-related GTP binding C	NM_022157	< 0.01	2.1844
IMPAD1	inositol monophosphatase domain containing 1	NM_017813	< 0.01	2.18558
SERPINB1	serpin peptidase inhibitor, clade B (ovalbumin), member 1	NM_030666	< 0.05	2.18697
MPZL1	myelin protein zero-like 1	NM_001146191 / NM_003953 / NM_024569	< 0.001	2.1872
PDCD2	programmed cell death 2	NM_002598 / NM_144781	< 0.01	2.19003
PPP1R14A	protein phosphatase 1, regulatory (inhibitor) subunit 14A	NM_033256	< 0.05	2.19066
TROAP	trophinin associated protein (tastin)	NM_001100620 / NM_005480	< 0.05	2.192
C15orf23	chromosome 15 open reading frame 23	NM_001142761 / NM_001142762 / NM_033286	< 0.01	2.19276
NPM1	nucleophosmin (nucleolar phosphoprotein B23, numatrin)	NM_001037738 / NM_002520 / NM_199185	< 0.01	2.19284
SPA17	sperm autoantigenic protein 17	NM_017425	< 0.05	2.19296

NCAPH	non-SMC condensin I complex, subunit H	NM_015341	< 0.01	2.19355
RALA	v-ral simian leukemia viral oncogene homolog A (ras related)	NM_005402	< 0.001	2.19395
TAP1	transporter 1, ATP-binding cassette, sub-family B (MDR/TAP)	NM_000593	< 0.05	2.19715
GLUL	glutamate-ammonia ligase	NM_001033044 / NM_001033056 / NM_002065	< 0.05	2.19868
H2AFV	H2A histone family, member V	NM_012412 / NM_138635 / NM_201436 / NM_201516 / NM_201517	< 0.001	2.20049
EIF4A2	eukaryotic translation initiation factor 4A2	NM_001967	< 0.001	2.20096
DPH3	DPH3, KTI11 homolog (S. cerevisiae)	NM_001047434 / NM_206831	< 0.01	2.2013
RNASEH2A	ribonuclease H2, subunit A	NM_006397	< 0.01	2.20202
C1orf9	chromosome 1 open reading frame 9	NM_014283 / NM_016227	< 0.001	2.20246
GLA	galactosidase, alpha	NM_000169	< 0.001	2.20252
FIGNL1	fidgetin-like 1	NM_001042762 / NM_022116	< 0.05	2.20272
SYT11	synaptotagmin XI	NM_152280	< 0.05	2.20286
CCDC88A	coiled-coil domain containing 88A	NM_001135597 / NM_018084	< 0.001	2.20557
ATL3	atlastin GTPase 3	NM_015459	< 0.05	2.20656
TAGLN2	transgelin 2	NM_003564	< 0.001	2.20892
DCAF13	DDB1 and CUL4 associated factor 13	NM_015420	< 0.05	2.21193
SLC45A4	solute carrier family 45, member 4	NM_001080431	< 0.05	2.21659
SGOL2	shugoshin-like 2 (S. pombe)	NM_001160033 / NM_001160046 / NM_152524	< 0.05	2.21807
MCM8	minichromosome maintenance complex component 8	NM_032485 / NM_182802	< 0.01	2.21906
CPSF6	cleavage and polyadenylation specific factor 6, 68kDa	NM_007007	< 0.001	2.22018
EDARADD	EDAR-associated death domain /// enolase 1, (alpha)	NM_001428 / NM_080738 / NM_145861	< 0.05	2.22069
C8orf76	chromosome 8 open reading frame 76	NM_032847	< 0.01	2.22088
ATAD2	ATPase family, AAA domain containing 2	NM_014109	< 0.05	2.22117
MRAS	muscle RAS oncogene homolog	NM_001085049 / NM_012219	< 0.01	2.22141
EED	embryonic ectoderm development	NM_003797 / NM_152991	< 0.001	2.22178

WBP5	WW domain binding protein 5	NM_001006612 / NM_001006613 / NM_001006614 / NM_016303	< 0.05	2.22203
VOPP1	vesicular, overexpressed in cancer, prosurvival protein 1	NM_030796	< 0.05	2.22584
ARL8B	ADP-ribosylation factor-like 8B	NM_018184	< 0.01	2.22601
MCTP1	multiple C2 domains, transmembrane 1	NM_001002796 / NM_024717	< 0.05	2.22613
RNF4	ring finger protein 4	NM_001185009 / NM_001185010 / NM_002938	< 0.001	2.2267
YWHAZ	tyrosine 3-monooxygenase/ tryptophan 5-monooxygenase activation protein, zeta polypeptid	NM_001135699 / NM_001135700 / NM_001135701 / NM_001135702 / NM_003406 / NM_14	< 0.001	2.22689

Note: There is a portion of up-regulation genes showed in the table.

Supplementary Table S3: Correlations of HIF-1α protein with clinicopathological characters

Variable	HIF-1α expression		*P* value
	Low	High	
Age (Years)			
≤ 60	61	58	0.369
> 60	10	14	
Sex			
Male	63	67	0.391
Female	8	5	
Preoperative HBV DNA, copies/mL			
$< 10^4$	33	38	0.451
$\geq 10^4$	38	34	
HBs Ag			
Negative	5	6	0.772
Postive	66	66	
HBe Ag			
Negative	52	49	0.496
Postive	19	23	
Liver cirrhosis			
No	18	8	0.027
Yes	53	64	
Ascites			
No	67	68	0.984
Yes	4	4	
Preoperative AFP (ng/ml)			
≤ 20	21	22	0.899
> 20	50	50	

Preoperative ALT(U/L)			
≤ 40	42	43	0.945
> 40	29	29	
Tumor diameter, cm			
≤ 5	34	17	0.002
> 5	37	55	
Tumor number			
Single	62	63	0.975
Multiple	9	9	
Tumor encapsulation			
Absence + incomplete	41	42	0.943
Complete	30	30	
Satellite nodules			
No	55	42	0.014
Yes	16	30	
Microscopic vascular invasion			
No	45	30	0.009
Yes	26	42	
Portal vein tumor thrombi			
No	70	60	0.002
Yes	1	12	
Tumor differentiation			
I + II	31	17	0.011
III + IV	40	55	
BCLC stage			
A	66	58	0.029
B + C	5	14	
TNM stage			
I	67	60	0.036
I + II	4	12	

Supplementary Table S4: Correlations of 14-3-3ζ protein with clinicopathological characters

Variable	14-3-3ζ expression		*P* value
	Low	High	
Age (Years)			
≤ 60	59	60	0.97
> 60	12	12	
Sex			
Male	64	66	0.751
Female	7	6	
Preoperative HBV DNA, copies/mL			
$< 10^4$	30	41	0.079
$\geq 10^4$	41	31	
HBs Ag			
Negative	3	8	0.122
Postive	68	64	
HBe Ag			
Negative	25	17	0.128
Postive	46	55	
Liver cirrhosis			
No	15	11	0.365
Yes	56	61	
Ascites			
No	68	67	0.479
Yes	3	5	
Preoperative AFP(ng/ml)			
≤ 20	21	22	0.899
> 20	50	50	
Preoperative ALT(U/L)			
≤ 40	41	44	0.682
> 40	30	28	
Tumor diameter, cm			
≤ 5	22	11	0.002
> 5	49	61	
Tumor number			
Single	63	62	0.637
Multiple	8	10	
Tumor encapsulation			
Absence + incomplete	38	45	0.277
Complete	33	27	
Satellite nodules			
No	50	45	0.316
Yes	21	27	
Microscopic vascular invasion			
No	44	31	0.024
Yes	27	41	

Portal vein tumor thrombus			
No	68	62	0.044
Yes	3	10	
Tumor differentiation			
I + II	26	22	0.443
III + IV	45	50	
BCLC stage			
A	67	57	0.007
B + C	4	15	
TNM stage			
I	68	59	0.009
II + III	3	13	

Note: The χ^2 test was used for comparison between groups.

Supplementary Table S5: Relationship between 14-3-3ζ expression and survival time

Time (month)	Relative 14-3-3ζ expression		*p* value
	Low	High	
Overall survival time	> 33	28	< 0.001$
Time to recurrence	12	24	< 0.001$

NOTE: Time to recurrence is defined as a period from surgery day to the time of tumor recurrence, the diagnosis of which is based on the typical feature presented in a CT/MRI scan and an elevated serum alpha-fetal protein.
$ Log-rank test.

Supplementary Table S6: Summary of clinicopathologic variables

Features	Values/Counts
Age (yrs),median (range)	49.47 (21–70)
Sex (male/female)	130/13
HBV DNA (IU/ml), median (range)	3 (0–32000)
HBVAg (positive/negative)	132/11
HBeAg (positive/negative)	42/101
Liver cirrhosis (yes/no)	117/26
Ascites (yes/no)	8/135
AFP (ng/ml),median (range)	149.3 (0.6–1210)
ALT (U/L, mean ± SD)	52.71 ± 50.54
Tumor diameter (cm, mean ± SD)	6.53 ± 3.69
Tumor number (single/multiple)	125/18
Encapsulation (complete/no)	83/60
Microstatelliate nodules (yes/no)	46/97
Microscopic vascular invasion (yes/no)	68/75
PVTT (yes/no)	13/130
Tumor differentiation (I + II/III + IV)	48/95
BCLC stage (A/B + C)	124/19

Abbreviations: AFP, alpha-fetoprotein; HBV, hepatitis B surface antigen; HBs Ag, Hepatitis B surface antigen; HBe Ag, Hepatitis B e antigen; ALT, alanine aminotransferase;

Supplementary Table S7: Sequence of primers for quantitative RT-PCR

Primer	Sequence (5′ to 3′)
14-3-3ζ forward primer	AGCAGATGGCTCGAGAATACA
14-3-3ζ reverse primer	GAAGCATTGGGGATCAAGAA
HIF-1α forward primer	TCATCCAAGAAGCCCTAACG
HIF-1α reverse primer	TTGCTTTCTCTGAGCATTCTG
E-cadherin forward primer	GACCGGTGCAATCTTCAAAA
E-cadherin reverse primer	CAGGTCTCCTCTTGGCTCTG
FN1 forward primer	CAGTGGGAGACCTCGAGAAG
FN1 reverse primer	TCCCTCGGAACATCAGAAAC
Desmoplakin forward primer	ATCAAGCAGTCGGAGCAGTT
Desmoplakin reverse primer	CAGGATGTACTATTCTCGGCG
Zo-1 forward primer	GACGTTTCCCCACTCTGAAA
Zo-1 reverse primer	CCTTGACATTGAGATTGCCACCTA
Vimentin forward primer	TCATCGTGATGCTGAGAAGTTTCG
Vimentin reverse primer	GGAGTCCGCAGTCTTACGAG
Twist forward primer	TCTGGACCTGGTAGAGG
Twist reverse primer	CCTCCCTGTCAGATGAGGAC
Snail forward primer	CCAGGCTGAGGTATTCCTTG
Snai reverse primer	AGAGCACAGCAATGGAGGAA
18s forward primer	CGGCTACCACATCCAAGGAA
18s reverse primer	GCTGGAATTACCGCGGCT
β-actin forward primer	AATCGTGCGTGACATTAAGGAG
β-actin reverse primer	ACTGTGTTGGCGTACAGGTCTT

Supplementary Table S8: List of the primary antibodies used for western blot, immunohistochemistry and immunoprecipitation analysis

Protein	Antibody	Application	Specificity	Epitope mapping	Company
14-3-3ζ	Rabbit	WB	Polyclonal	Full length	Abcam
14-3-3ζ	Rabbit	IHC/Co-IP/IF	Polyclonal	Full length	Abcam
HIF-1α	Rabbit	WB	Polyclonal	Full length	Cell Signal Technology
HIF-1α	Rabbit	IHC/Co-IP/IF	Polyclonal	Full length	Abcam
E-cadherin	Rabbit	WB	Polyclonal	Full length	eBioscience
Vimentin	Mouse	WB/IHC	Monoclonal	Full length	eBioscience
Snail	Rabbit	WB	Polyclonal	Full length	Proteintech
HDAC4	Rabbit	WB/IF	Polyclonal	Full length	Abcam
VEGF	Rabbit	WB	Polyclonal	Full length	Cell Signal Technology
β-actin	Rabbit	WB	Monoclonal	Full length	Santa Cruz Technology

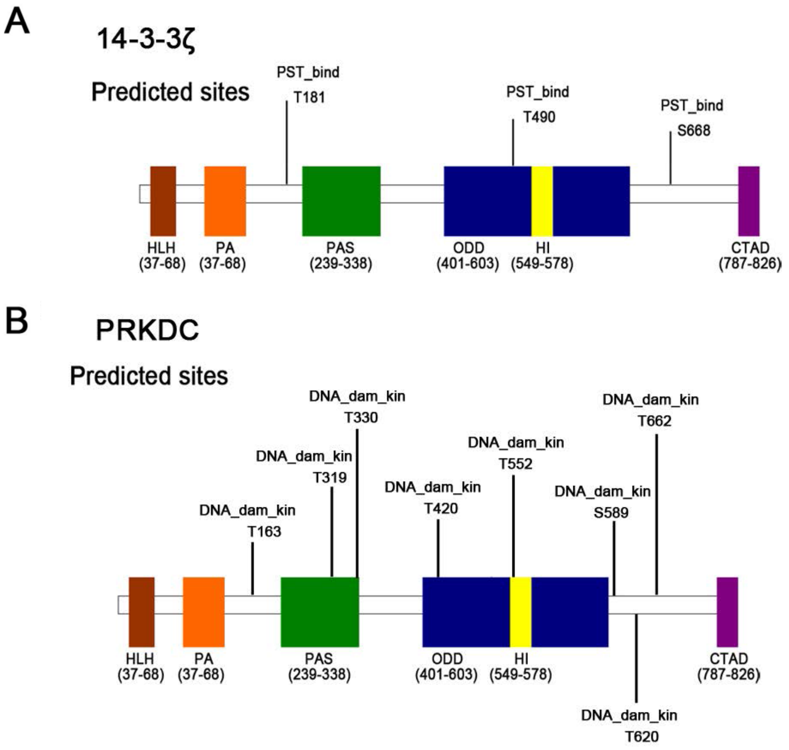

Supplementary Figure S1: The 14-3-3ζ (A) or PRKDC (B) binding sites on HIF-1α protein were predicted using Scansite software.

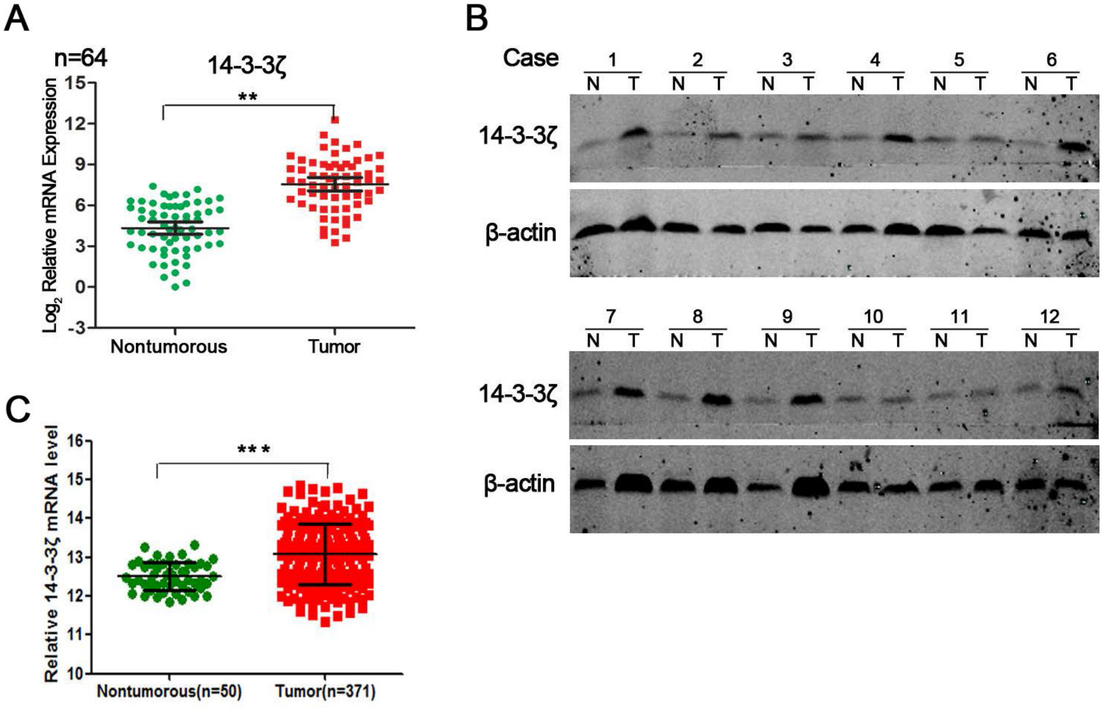

Supplementary Figure S2: (**A** and **B**) 14-3-3ζ expression in paired tumor tissues and nontumorous tissues was examined by quantitative real time PCR (A) and western blot (B). Data showed as means ± SEM. $^{**}P < 0.01$. (**C**) 14-3-3ζ expression analysis using published datasheet from Liver hepatocellular carcinoma (LIHC) in The Cancer Genome Atlas (TCGA) database. Data showed as means ± SEM. $^{***}P < 0.0001$.

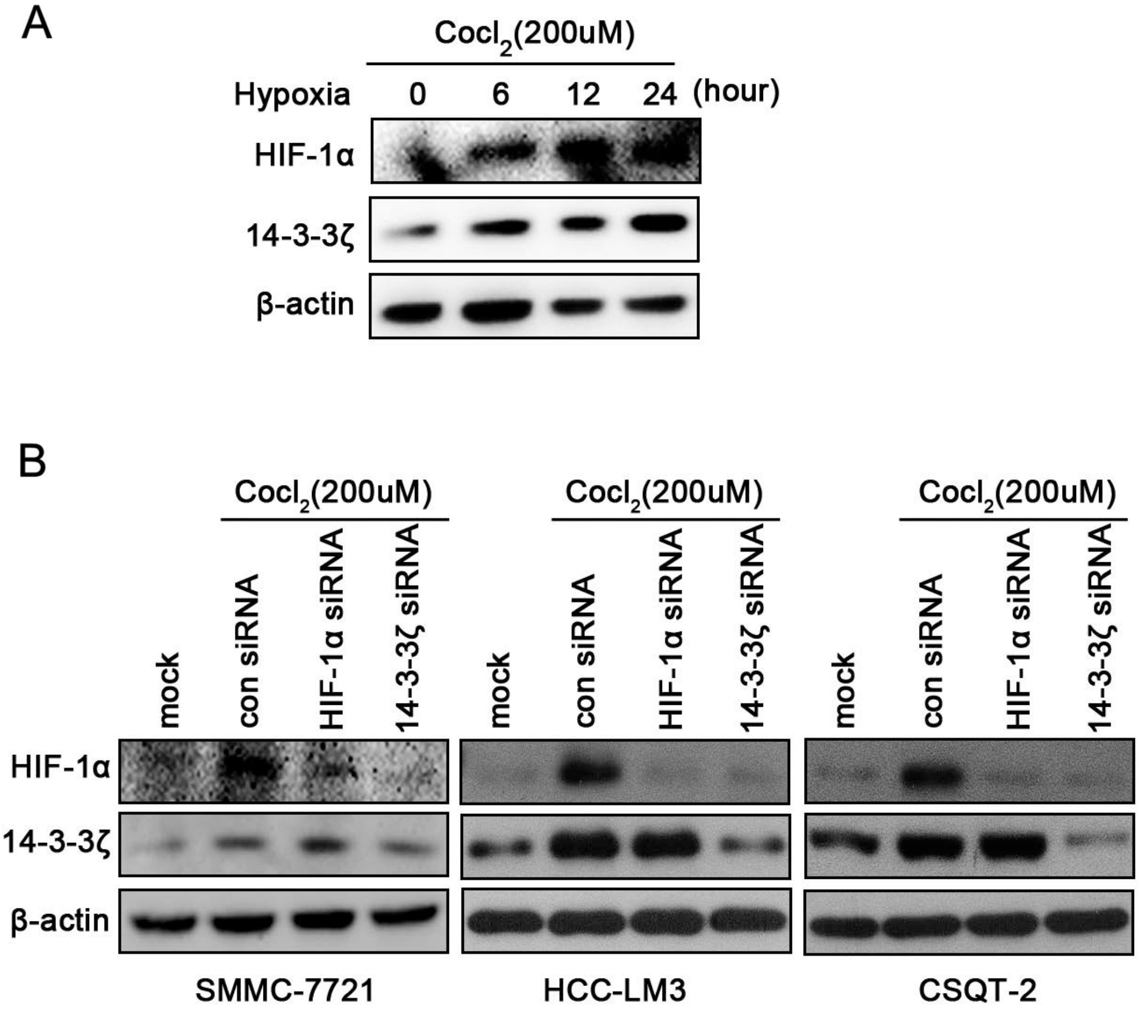

Supplementary Figure S3: (**A**) SMMC-7721 cells treated with CoCl2 for different time, then 14-3-3ζ and HIF-1α proteins were evaluated using western blot. (**B**) SMMC-7721, HCC-LM3 and CSQT-2 cells were treated with siRNAs to block HIF-1α and 14-3-3ζ expression and were then incubated in the presence of CoCl2 for 18 hours. HIF-1α and 14-3-3ζ proteins were detected using western blot.

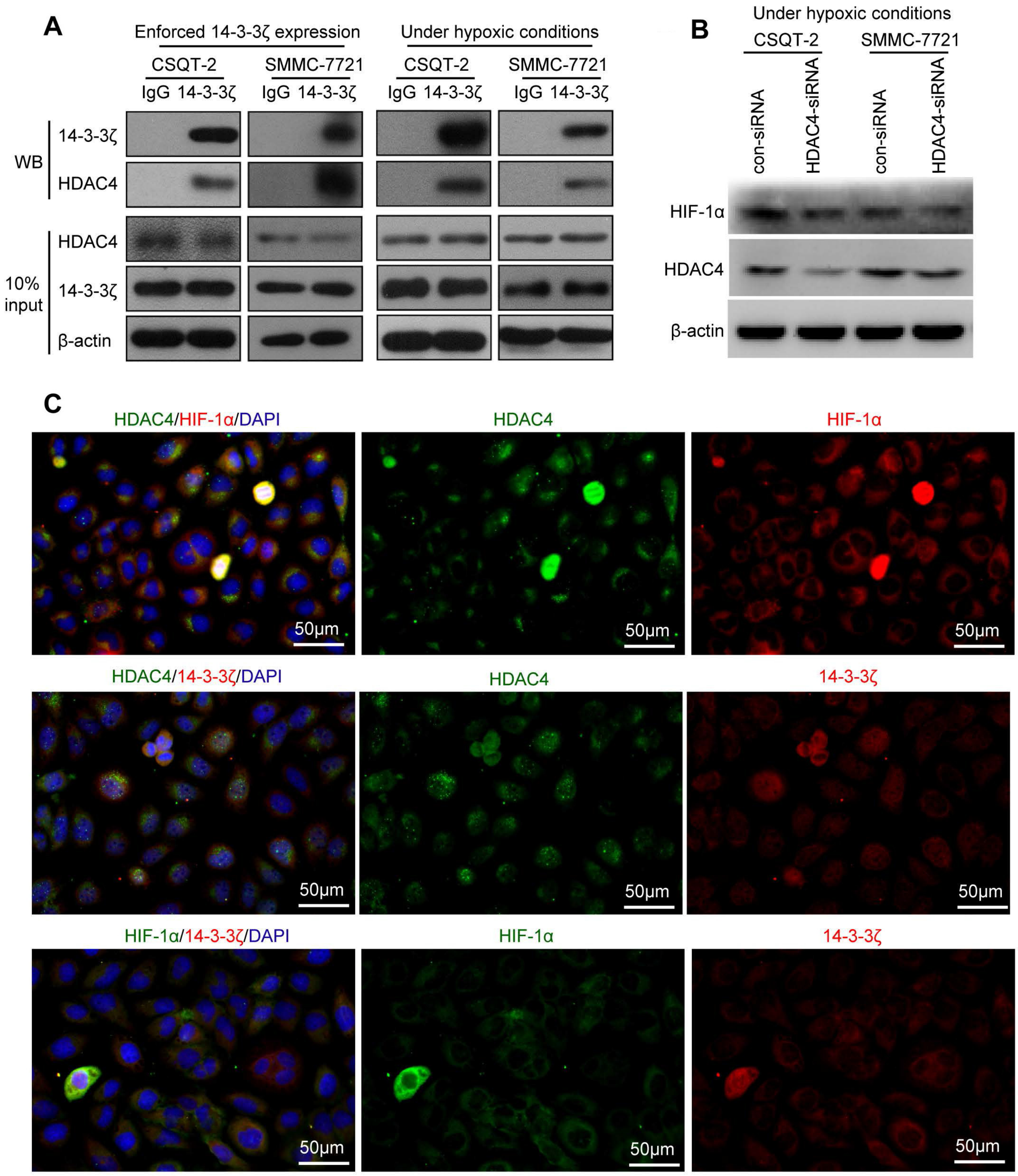

Supplementary Figure S4: (**A**) The interaction between 14-3-3ζ and HDAC4 was determined by co-IP assays in SMMC-7721 and CSQT-2, which have been forced 14-3-3ζ expression or treated with CoCl2. (**B**) Down-regulation of HDAC4 by HDAC4 shRNA decreased HIF-1α protein. (**C**) Co-localization of HDAC4/HIF-1α, HDAC4/14-3-3 and HIF-1α/14-3-3 in SMMC-7721 cells under hypoxic condition analyzed by IF. Bars, 50 μm.

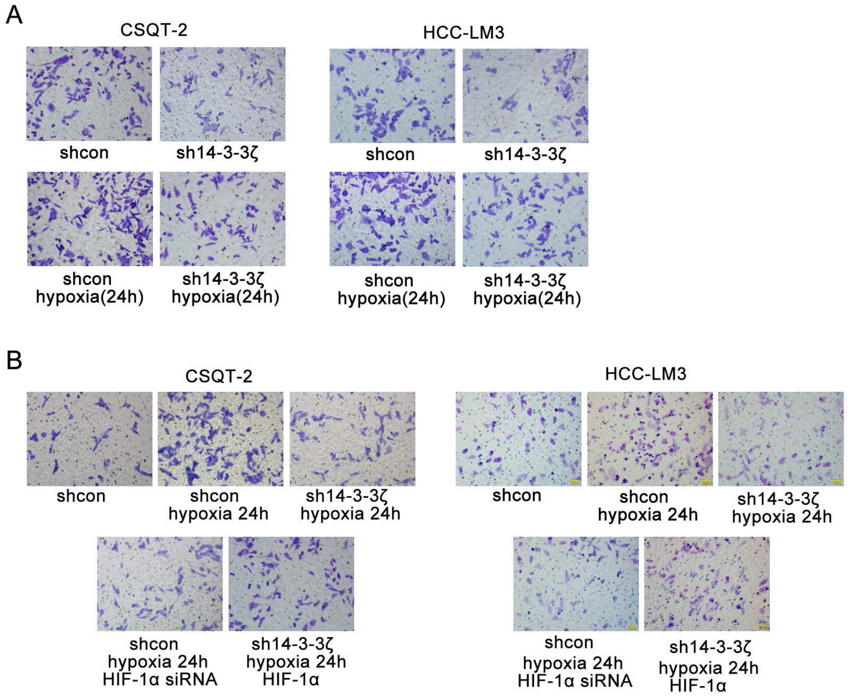

Supplementary Figure S5: (A) Under normoxic/hypoxic conditions, the aggressive behavior of CSQT-2 shcon/sh14-3-3ζ cells and HCC-LM3 shcon/sh14-3-3ζ cells were analyzed by way of the invasion assay using a matrigel-coated boyden chamber. The representative images are showed. (**B**) The invasion assays were performed for the indicated cells following treatment with or without ectopic HIF-1α expression. Cell invasion was determined with crystal violet staining. The representative images are showed.

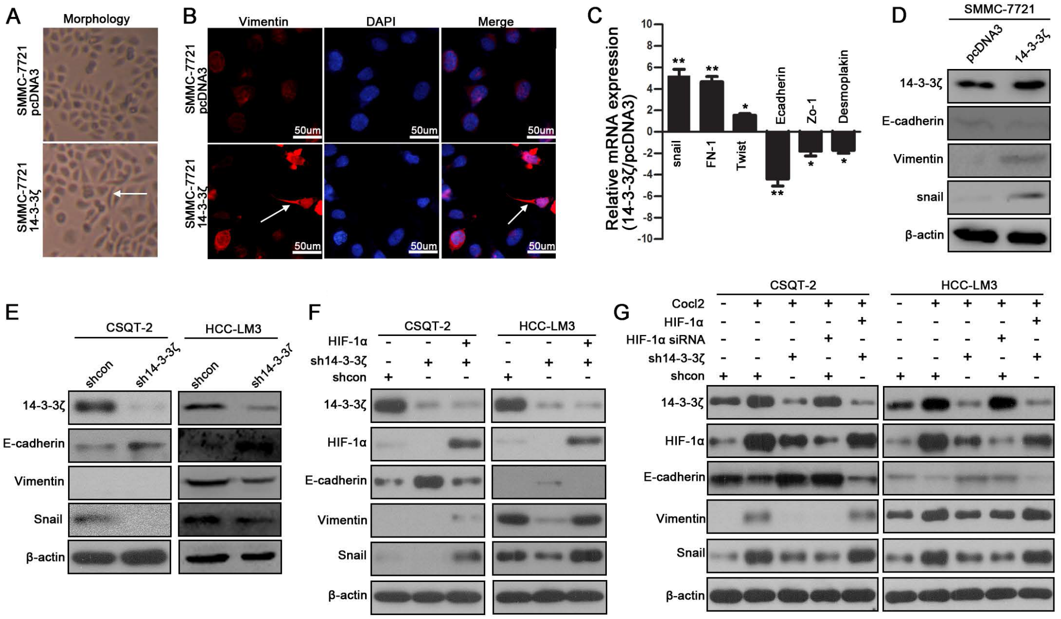

Supplementary Figure S6: (A) Representative image of SMMC-7721 cells with (SMMC-7721/14-3-3ζ) or without (SMMC-7721/pcDNA3) 14-3-3ζ over-expression. (**B**) Representative image of Vimentin Immunofluorescence staining in SMMC-7721 cells with (SMMC-7721/14-3-3ζ) or without (SMMC-7721/pcDNA3) 14-3-3ζ over-expression. (**C**) The expression of epithelial markers (E-cadherin, ZO-1 and desmoplakin) and mesenchymal markers (snail, fibronectin and twist) were determined using real-time PCR in SMMC-7721 cells with (SMMC-7721/14-3-3ζ) or without (SMMC-7721/pcDNA3) 14-3-3ζ over-expression. (**D**) Expression of E-cadherin, Vimentin and Snail were determined using western blot in SMMC-7721 cells with (SMMC-7721/14-3-3ζ) or without (SMMC-7721/pcDNA3) 14-3-3ζ over-expression. (**E**) Expression of E-cadherin, Vimentin and Snail were determined using western blot in CSQT-2 shcon/sh14-3-3ζ cells and HCC-LM3 shcon/sh14-3-3ζ cells. (**F**) Expression of E-cadherin, Vimentin and Snail were determined using western blot in the indicated cells following treatment with or without ectopic HIF-1α expression. (**G**) Under hypoxic conditions, expression of E-cadherin, Vimentin and Snail were determined using western blot in the indicated cells following treatment with HIF-1α siRNA or ectopic HIF-1α expression.

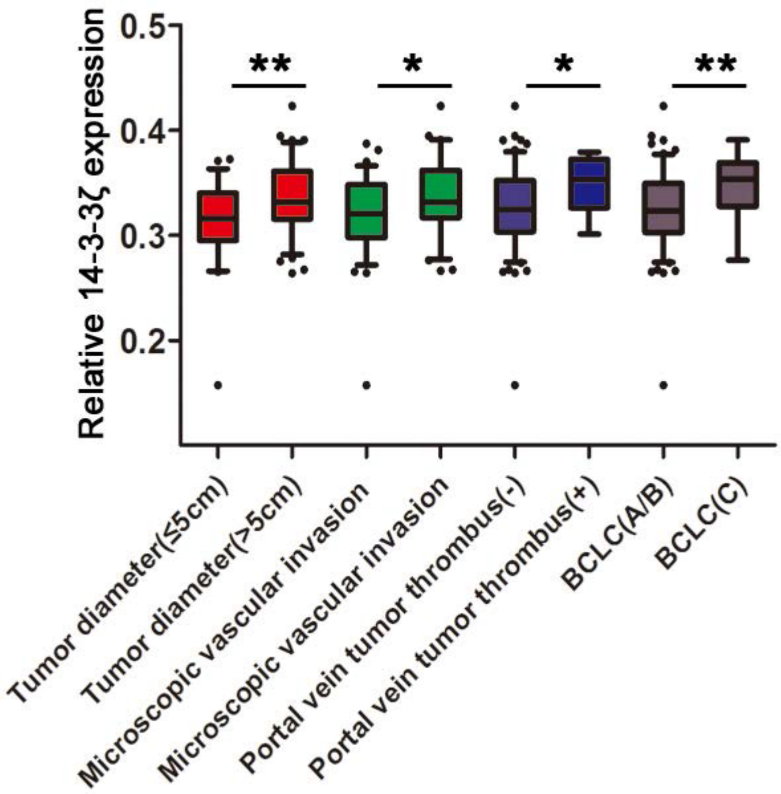

Supplementary Figure S7: Relative 14-3-3ζ expression in 143 HCC patients with or without larger tumor (> 5 cm), microscopic vascular invasion, PVTT, advanced stage. Date showed as means ± SEM. **P* < 0.05 and ***P* < 0.01.

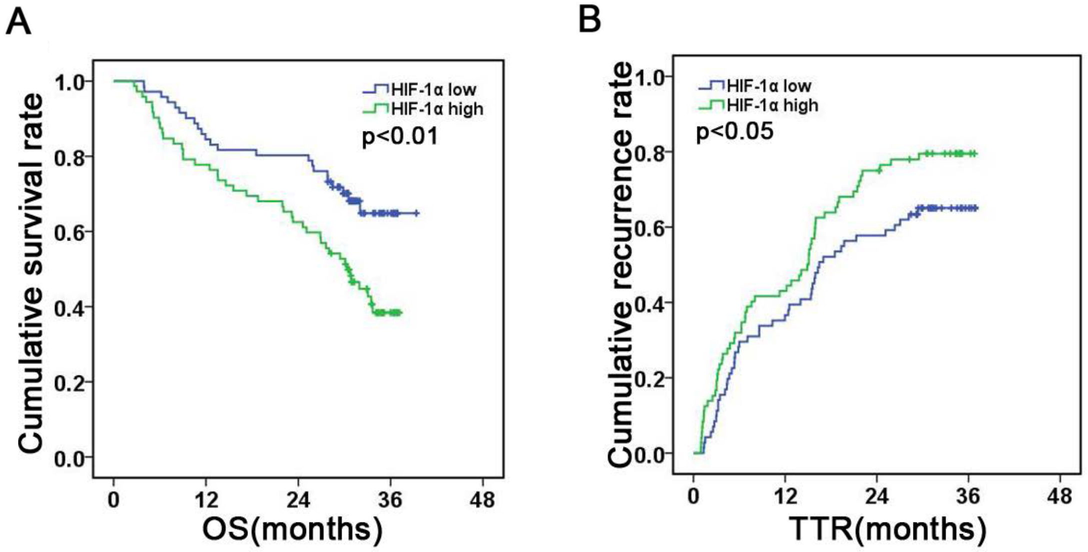

Supplementary Figure S8: The cumulative survival (A) and recurrence rate (B) were analyze in HIF-1α low /high group of HCC patients (the two-sided long-rank test).
